# Bedside to bench: the outlook for psychedelic research

**DOI:** 10.3389/fphar.2023.1240295

**Published:** 2023-10-02

**Authors:** Victor P. Acero, Emily S. Cribas, Kevin D. Browne, Olivia Rivellini, Justin C. Burrell, John C. O’Donnell, Suradip Das, D. Kacy Cullen

**Affiliations:** ^1^ Center for Brain Injury and Repair, Department of Neurosurgery, Perelman School of Medicine, University of Pennsylvania, Philadelphia, PA, United States; ^2^ Center for Neurotrauma, Neurodegeneration and Restoration, Corporal Michael J. Crescenz Veterans Affairs Medical Center, Philadelphia, PA, United States; ^3^ Department of Bioengineering, School of Engineering and Applied Science, University of Pennsylvania, Philadelphia, PA, United States; ^4^ Penn Psychedelics Collaborative, University of Pennsylvania, Philadelphia, PA, United States; ^5^ Department of Microbiology, Perelman School of Medicine, University of Pennsylvania, Philadelphia, PA, United States

**Keywords:** psychedelics, psilocybin, salvinorin, ketamine, mechanism of action (MOA), MDMA, DMT, ayahuasca

## Abstract

There has recently been a resurgence of interest in psychedelic compounds based on studies demonstrating their potential therapeutic applications in treating post-traumatic stress disorder, substance abuse disorders, and treatment-resistant depression. Despite promising efficacy observed in some clinical trials, the full range of biological effects and mechanism(s) of action of these compounds have yet to be fully established. Indeed, most studies to date have focused on assessing the psychological mechanisms of psychedelics, often neglecting the non-psychological modes of action. However, it is important to understand that psychedelics may mediate their therapeutic effects through multi-faceted mechanisms, such as the modulation of brain network activity, neuronal plasticity, neuroendocrine function, glial cell regulation, epigenetic processes, and the gut-brain axis. This review provides a framework supporting the implementation of a multi-faceted approach, incorporating *in silico*, *in vitro* and *in vivo* modeling, to aid in the comprehensive understanding of the physiological effects of psychedelics and their potential for clinical application beyond the treatment of psychiatric disorders. We also provide an overview of the literature supporting the potential utility of psychedelics for the treatment of brain injury (e.g., stroke and traumatic brain injury), neurodegenerative diseases (e.g., Parkinson’s and Alzheimer’s diseases), and gut-brain axis dysfunction associated with psychiatric disorders (e.g., generalized anxiety disorder and major depressive disorder). To move the field forward, we outline advantageous experimental frameworks to explore these and other novel applications for psychedelics.

## 1 Introduction

Psychedelics refer to a diverse array of chemical compounds that induce distinctly “mystical-type” experiences and non-ordinary states of consciousness. The term “psychedelic”, meaning mind manifesting, was introduced by Humphrey Osmond in 1956, as an alternative to “psychomimetic”, meaning “madness mimicking” (Lee, 1992; Dyck, 2006). Some scholars also proposed the term “entactogen”, meaning *touching within* or *reaching inside*, due to the capacity for certain compounds to enhance therapeutic memory retrieval. The 1950s and 1960s would see an explosion in research into the therapeutic potential and psychopharmacological characteristics of plant-based and synthetic psychedelic compounds. Unfortunately, all biomedical and scientific research into these psychedelic substances ceased upon passage of the 1970 Controlled Substances Act, which classified these compounds as Schedule I, the category reserved for the most dangerous and abusable of all illicit drugs. However, in recent years, grassroots efforts and shifting public opinion have led to these ancient medicines being once again objects of intensive scientific scrutiny and their clinical potential is being explored.

For instance, randomized double-blind clinical trials have shown psychedelic-assisted therapy to be highly efficacious in treating treatment-resistant depression (TRD), major depressive disorder (MDD) post-traumatic stress disorder (PTSD), and substance abuse disorders (SUD) (Mithoefer et al., 2019; Carhart-Harris1 et al., 2018; Noorani et al., 2018; Gasser et al., 2014). Of note, 3,4-Methyl enedioxymethamphetamine (MDMA) in MDMA-assisted psychotherapy for the treatment of moderate-to-severe PTSD was found to cause symptom remission or reduction that persisted for up to 3.5 years after therapy (Mithoefer et al., 2013). The recently published phase 3 clinical trial results for MDMA-assisted therapy for PTSD showed that 67% of patients no longer met the PTSD criteria, 21% had a clinically significant response, and 12% had no response, while the placebo with therapy group had 32% of patients no longer meeting the PTSD criteria, 30% with a clinically meaningful response, and 30% with no response (Mitchell et al., 2021). The remarkable clinical efficacy has culminated in the opening of multi-million-dollar research centers to investigate therapeutic applications of psychedelics at Johns Hopkins University, Imperial College London, and University of California-Berkeley.

Despite this recent surge in psychedelic research, the full range of effects and biological mechanisms of action are far from understood (Garcia-Romeu et al., 2016; Flanagan and Nichols, 2018). Most clinical research has been focused on assessing the psychological–rather than basic physiological–mechanisms of psychedelics. Given these compounds ability to induce transformative and personally meaningful experiences, which correlate with positive behavioral changes, there is a tendency to disregard how psychedelics modulate physiological processes. Psychological wellbeing, however, is interwoven with various physiological systems, e.g., proper neuroendocrine and inflammatory function. Understanding the non-psychological effects of psychedelics may also allow for development of a range of novel clinical applications. We suggest that such foundational investigations will require multi-disciplinary investigations which leverage the collective strengths of *in silico*, *in vitro*, *in vivo* models of homeostatic as well as injury or disease-based conditions.

## 2 Current trends in psychedelic research

Plant medicines containing psychedelic compounds, such as ayahuasca, peyote, psilocybin mushrooms, played a central spiritual and medicinal role in indigenous cultures for millennia (Naples, 2013; Armijos et al., 2014; Carod-Artal, 2015; Jay, 2019; Arie et al., 2020). Psychedelics do not have a universally shared pharmacological action or chemical structure, and are often defined by the profound and meaningful subjective psychedelic experiences they induce, e.g., a dissolution of the *ego*, a “*mystical”* phenomenological experience, and impairment of spatiotemporal processing (Krupitsky et al., 2002; Dakwar et al., 2014; Zanos et al., 2018). However, these experiences can also be induced by non-psychedelic compounds, such as the fast-acting antidepressant dissociative ketamine. Thus, rather than a strict all-encompassing definition, the categorization of these diverse drugs as “psychedelics” is somewhat arbitrary.

Psychedelics may be organized into distinct classes based on their structural characteristics: 1) *tryptamines*, such as psilocin (the active form of psilocybin) and N,N-dimethyltryptamine (DMT); 2) *lysergamides*, such as lysergic acid diethylamide (LSD); 3) *phenethylamines*, including 3,4-Methyl enedioxymethamphetamine (MDMA) and 2,5-dimethoxy-4-iodoamphetamine (DOI); 4) *cannabinoids* like *Δ*-9-tetrahydrocannabinol*;* and 5) *terpenoids*, such as Salvinorin A (Szabo, 2015). Although, psychedelics may also be categorized by their pharmacological action (serotonergic, glutamatergic, dopaminergic, etc.), they often have a complex pharmacological profile. Ultimately, action on a single receptor type cannot adequately explain their acute or chronic effects (Garcia-Romeu et al., 2016; Zamberlan et al., 2018). Further research into psychedelic compounds and the mechanisms that mediate their clinical efficacy may eventually allow for a more meaningful classification schema.

### 2.1 Need for preclinical and mechanistic studies

Although psychedelics have great therapeutic promise, the psychophysiological theories explaining how psychedelics manifest their clinical effects are nascent. Research leveraging brain imaging techniques has played a significant role in addressing this gap in knowledge. For example, it appears that characteristics of the psychedelic experience are constructed by a combination of diminished top-down modulation of sensory processing, an increase in the total content of one’s sensorium, and functional disintegration of distributed networks. Carhart-Harris et al. formulated the Relaxed Brain Under Psychedelics (REBUS) model of psychological annealing under psychedelics, which posits that psychedelics induce a state of neuropsychological plasticity under which embedded cognitive biases can be re-written. This a framework helps explain why psychedelics can aid in treatment of such a wide array of psychiatric disorders (Carhart-Harris and Friston, 2019).

While human brain research, particularly neuroimaging, has been instrumental in advancing our understanding of the psychedelic experience, identification of novel therapeutic targets, and understanding possible modes of action. Undeniably, the main strength of human neuroimaging research is that it allows for identification of neural correlates of specific elements of the subjective experience, such as those assessed by the mystical experience questionnaire, which are impossible to isolate in animal models. However, human neuroimaging research has its limits. For instance, there is huge variability in how studies are conducted, i.e., “eyes-closed” or “eyes-open”, and only a few resting state fMRI datasets have laid the foundation for data processing and analysis methodology (McCulloch et al., 2022). Moreover, the small sample sizes and lack of standards for the field makes it difficult to pool together datasets in order to generate larger sample sizes necessary for reproducible brain-wide association studies (Marek et al., 2022). Moreover, it is highly correlational due to its low spatiotemporal resolution and non-ideal for robust probing of cellular mechanisms. This is where *in vitro* and *in vivo* research comes into play–these experimental approaches allow for more high-throughput, mechanistic understanding of how psychedelics effect neuronal circuits and cellular processes, and thus compliment the insights derived from human neuroimaging studies.

Historically, for a diverse class of compounds, robust preclinical *in vitro* and *in vivo* research has been vital for establishing a foundational pharmacological understanding and will therefore be crucial in advancing our understanding of psychedelics. For instance, animal models permit invasive measurements that are impossible with humans, yet this has been poorly leveraged in the field. Specifically, *in vitro* or *in vivo* electrophysiology studies using techniques with high spatiotemporal resolution could be utilized to explore characterized processes for mediating spatial and temporal processing. For instance, *Domenico et al.* found LSD impaired communication between cortical sensory circuits and hippocampal place cells, and triggered oscillatory activity which naturally occurs during wakefulness-to-sleep transitions (Domenico et al., 2021). This helps further understand the neurological substrate for hallucinatory experiences. Extending beyond the realm of neuroscience, animal models have also contributed to our understanding of the broader pharmacological properties of psychedelic compounds. For instance, rodent models of allergic asthma were utilized to screen serotonergic compounds for maximal anti-inflammatory effects and elucidate the pathways necessary for anti-inflammatory action in lung tissue (Flanagan et al., 2021). While mammalian models are traditionally used for assessments of cellular and molecular mechanisms, there are alternative high-throughput models that would be ideal for preliminary screening and characterization of novel psychedelic compounds in healthy and diseased individuals. For instance, the zebra fish (*Danio rerio*) model has a fully characterized genome and nervous system, is low-cost and high throughput, and has high physiological homology to humans (Neelkantan et al., 2013). Moreover, the response of this model to various psychedelic compounds has been well characterized (Neelkantan et al., 2013).

Furthermore, due to regulatory and financial barriers to psychedelic research, only a relatively narrow range of compounds have been thoroughly investigated. In fact, Dr. Alexander Shulgin’s *PiHKAL: A Chemical Love Story* is an astonishing chemical catalog of over 170 novel psychedelic compounds (including MDMA) which has been largely ignored across *in vitro, in vivo*, and, especially, human studies (Dean et al., 2013). While there are *in vivo* studies involving rodent or zebrafish models, these studies often only assess effects of non-scheduled compounds like 2,5-dimethoxy-4-iodamphetamine (DOI). Furthermore, most studies lack informative comparisons across different psychedelics, often due to the difficulty of acquiring multiple compounds. However, there are some landmark studies, e.g., which conduct robust comparisons across various psychedelic compounds with distinct pharmacological profiles. Such studies include Ly et al. (2018) that provided a robust characterization of the plasticity promoting effects of psychedelics and the cellular signaling pathways which mediating these effects, and Nardou et al. (2023) which demonstrated the capacity of psychedelics to open a social critical period by mediated metaplastic restoration of oxytocin-mediated long-term depression. While such studies have robust cross-drug comparisons, they often focus on otherwise well studied compounds (e.g., LSD, psilocybin, MDMA, ketamine, ibogaine), and there still remains a need to examine understudied classes of psychedelic compounds, such as the 2C series drugs (ring-substituted phenethylamines). It could be advantageous to expand our investigation of psychedelics to include more compounds in order to build a robust understanding of their effects and mechanisms, and discover more nuanced therapeutic applications for distinct compounds.

The utility of *in silico* research, i.e., computational models and simulations, in psychedelic research has become increasingly evident. A noteworthy example of the application of computational models is the Wacker et al study that revealed the crystal structure of an LSD-bond human 5-HT_2A_ receptor using molecular dynamics smulations (Wacker et al., 2017). This work delineated the active-state structure of the bound receptor, providing crucial insights into the molecular mechanisms underlying psychedelic actions, thereby potentially accelerating drug discovery for a variety of novel compounds as well (Wacker et al., 2017). Moreover, recent advancements in artificial intelligence (AI) and virtual reality (VR) technology are increasing the functionality and utility of *in silico* approaches for psychedelic research. By utilizing predictive models based on the structure-activity relationship (SAR) of known psychedelic molecules, AI algorithms can generate new compounds with improved pharmacological properties and reduced side effects. Such techniques are especially useful in processing large datasets, which is an essential aspect of drug discovery. Such tools are already being utilized by numerous drug companies (e.g., Enveric Biosciences, April19 Discovery and PharmaTher) to help identify novel psychedelic compounds with minimal adverse effects and maximal therapeutic benefits. Moreover, analyzing large datasets of clinical and preclinical studies, machine learning algorithms can identify patterns of treatment response and predict the optimal dosing regimens for specific patient populations. Also, the emergence of VR platforms for medicinal chemistry, e.g., Nanome, has allowed for a 3D visualization of molecular (crystal-based and ligand-based) structures which captures details often lost in 2D visualizations (Zhavoronkov et al., 2020). As psychedelic research expands and these emerging technologies mature, *in silico* methodologies will likely assume an increasingly vital role in propelling drug discovery and development endeavours.

In writing this review, we have three parallel objectives: 1) provide a review of potential modes of action that underlie observed psychedelic effects, 2) to propose the re-centering of *in vitro* and *in vivo* models as cornerstones to understanding psychedelics and their therapeutic applications, and 3) to propose novel therapeutic applications of psychedelics and relevant models for investigating these applications. To accomplish these objectives, we will first review recent literature regarding the major clinical applications of psychedelics. Second, we will discuss what is known about the modes of action for psychedelic drugs. Finally, we propose novel directions for psychedelic research, and relevant *in vivo* and *in vitro* research models that may complement future investigations.

### 2.2 Clinical use of psychedelics

Modern clinical use of psychedelics began with LSD- and psilocybin-assisted therapy targeted at alleviating existential distress, depression, and anxiety in patients with life-threatening illnesses (Gasser et al., 2014; Ross et al., 2016). In fact, it was the first pharmacological intervention for “existential distress” (e.g., a psychological state characterized by feelings of anxiety, helplessness, loneliness, and loss of meaning, often stemming from contemplation of one’s mortality) making it an attractive tool in oncology and palliative care. More importantly, these studies demonstrated the safety of psychedelic administration within clinical settings, specifically for alleviation of psychological suffering. Since then, psychedelics have continued to occupy a spotlight in neuropsychopharmacology by producing similarly robust and long-lasting benefits in the treatment of SUD, MDD, and PTSD. Interestingly, the efficacy of psychedelic-assisted therapy was significantly correlated with the personal meaningfulness and occurrence of a “mystical experience” (Gasser et al., 2014; Griffiths et al., 2016; Ross et al., 2016). Yet, as is the case with most psychedelic clinical targets, it is unclear to what extent the clinical efficacy is mediated by bottom-up neurobiological modulation as opposed to a top-down cognitive mechanism. We will cover these clinical applications briefly, as they have already been thoroughly discussed in other reviews.

#### 2.2.1 Substance use disorders

SUD is a complex disorder with genetic, environmental, and psychosocial etiologies. It is characterized by persistent craving and/or continued and excessive use leading to dysfunctional behavior. While addiction research has helped uncover alterations to dopaminergic circuit function and reward signaling pathways, these alterations are not predictive of an individual’s capacity for relapse and have not led to meaningful therapeutic interventions (Hart, 2017; Miller et al., 2020). Studies utilizing psychedelic-assisted psychotherapy have shown promising results for improving recovery from this complex disease. Psychedelics like psilocybin, ibogaine, ayahuasca, and ketamine have been shown to facilitate abstinence from nicotine, opioids, alcohol, and cocaine respectively, as well as facilitate overall positive effects on anxiety, mood, and depression as well (Re et al., 2016; Brown and Alper, 2018; Jones et al., 2018; Noorani et al., 2018).

#### 2.2.2 Major depressive disorder and treatment resistant depression

MDD accounts for over 50% of psychiatric consultations and is characterized by persistent sadness and loss of interest. TRD describes an affliction of MDD that is non-responsive to anti-depressants or behavioral therapy. However, due to the lack of a clear definition - often interpreted as insufficient response to two antidepressant trials - estimates of its prevalence in the MDD population can vary widely. Clinical trials, as well as animal studies, have demonstrated multiple psychedelics, e.g., psilocybin and DMT, have fast-acting and long-lasting anti-depressant properties (Johnson et al., 2011; Robson et al., 2012; Palhano-Fontes et al., 2019). Notably, non-serotonergic psychedelics like ketamine demonstrate fast-acting, but short lived, anti-depressant effects. The recent Breakthrough Therapy designation of psilocybin for MDD and TRD by the FDA is a testament to its clinical potential. Of note, recent phase 2 double-blind trials for psilocybin-assisted psychotherapy for TRD found that large (25 mg) doses were able to produce significant remission in 29% and 20% participants at 3-week and 12-week post-treatment (Goodwin et al., 2022).

#### 2.2.3 Anxiety disorders

Pathological anxiety is a state of exaggerated hypervigilance and responsiveness to fearful stimuli and has various clinical manifestations. Anxiety disorders, including general anxiety disorder (GAD), social anxiety disorder (SAD), and PTSD, are amenable to psychedelic treatment. For instance, in phase 2 trials of MDMA-assisted psychotherapy for PTSD, 61% and 68% of participants at 2 months and 1-year follow-ups, respectively, no longer met PTSD diagnostic criteria (Fe et al., 2019; Mithoefer et al., 2019). Patients with remission have been reported to maintain remission for up to 6 years (Sessa et al., 2019). Notably, MDMA is a potent stimulator of serotonin norepinephrine and oxytocin release, which could underlie the reported prosocial effects, emotional regulation, and attenuation of amygdala response to anxiogenic stimuli (Morley and McGregor, 2000; Johansen and Krebs, 2009; Carhart-Harris et al., 2014a).

## 3 Psychedelic mode of action

**FIGURE 1:**
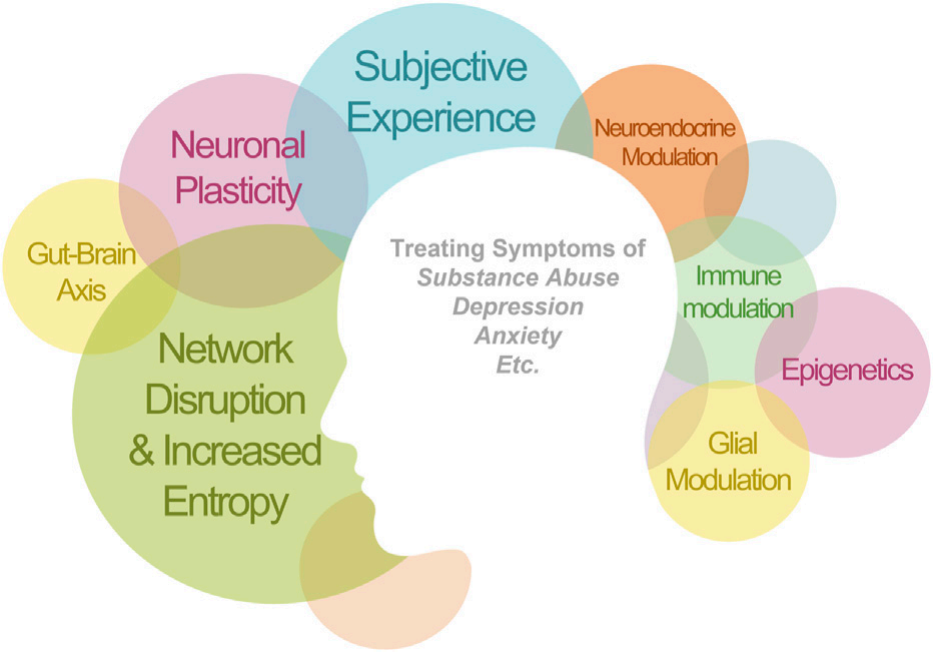
Therapeutic applications of psychedelics that have been demonstrated clinically. The clinical efficacy is likely mediated synergistically by multiple mechanisms of action. The size of each circle is proportional to the amount of evidence for this mechanism of action. There are circles left empty as a reminder for the yet validated mechanisms.

The novel nature of psychedelic drugs might help advance and reconstruct our paradigms for psychiatric disorders and the fundamental nature of consciousness. Likewise, we should be wary about condensing the therapeutic mode of action into a single mechanism. For instance, when neuroscience was a nascent discipline, scientists grossly overemphasized the role of neurons in brain function, due to their easy visualization and study using early staining techniques. However, we now know that glial cells–over 10-fold more numerous than neurons–play key roles in regulating brain function, homeostasis, remodeling, and perfusion, amongst many others. Likewise, it is necessary for us to examine psychedelic treatment through multiple scientific lenses in order to fully understand their effects. Ultimately, it is unlikely that a single mode of action can adequately account for the effects of psychedelics, instead, multi-faceted actions likely work synergistically across multiple systems to provide robust therapeutic effects (Figure 1).

Despite advancements in understanding the various mechanisms contributing to psychedelic therapeutic action, the significance of the subjective experience remains hotly contested (Olson, 2021; Yaden and Griffiths, 2021). Psychedelic-assisted psychotherapy often aims to cultivate the profoundly meaningful, psychologically transformative experiences that often precede major shifts in psychological wellbeing in patients. Various scholars have developed scales to quantify these experiences, which are often referred to as “mystical”, “peak”, “or “spiritual” (Zamberlan et al., 2018; Yaden and Newberg, 2022). For example, the Mystical Experience Questionnaire (MEQ), measures seven components of experience: 1) *internal of unity* (“merging with ultimate reality” or “pure awareness”); 2*) external unity* (“all is one” or “all things are alive”); 3) *transcendence of time and space*, 4) *ineffability and paradoxicality* (difficulty describing the experience in words); 5) *sense of sacredness* (awe); 6) *noetic quality* (intuitive knowledge of ultimate reality or self), 7) *deeply felt positive mood*. Those seeking an in-depth exploration can refer to Yaden and Newberg’s work, *The Varieties of Spiritual Experience*, which provides a thorough multidisciplinary review of the research (Yaden and Newberg, 2022)*.* Studies suggest that the subjective intensity and quality of peak psychedelic (or “*mystical*”) experience, e.g., ego death and increased sense of connection, is predictive of psychedelic-assisted therapy efficacy (Barrett et al., 2017; Prelle et al., 2017; Roseman et al., 2018; Swanson, 2018). In the Altered States of Consciousness (ASC) questionnaire, scores on the Oceanic Boundlessness (OBN) scale, which quantifies mystical states of mind, were notably more predictive of positive clinical outcomes for patients with treatment-resistant depression (TRD) than scores related to altered visual or auditory perception (Roseman et al., 2018). Further clarifying the role of the subjective experience, a recent cross-sectional survey study examining the effects of psychedelic experiences, it was found that both mystical-type experiences and psychological insights during the psychedelic experience were associated with subsequent decreases in depression and anxiety (Davis et al., 2015). Moreover, they demonstrated that increased psychological flexibility was a mediator in the relationship between these acute psychedelic effects and the observed decreases in depression and anxiety symptoms (Davis et al., 2015). Notably, insightful as well as mystical-type experiences have been identified as subtypes of the phenomenon of *quantum change*, i.e., “a sudden, distinctive, benevolent, and enduring experience resulting in personal transformations that affect a broad range of personal emotions, cognitions, and behavior” (Davis et al., 2015).

Moreover, non-psychedelic, or non-subjective, analogs of these drugs may provide insight into the clinical significance of the acute experience. 2-Br-LSD and tabernathalog, non-hallucinogenic analogs of LSD and ibogaine, respectively, have demonstrated anti-depressant and anti-addictive effects in rodents (Cameron et al., 2021; Lewis et al., 2023a). It remains to be seen whether these compounds would be efficacious or safe for psychiatric applications. For instance, lanicemine, a non-psychedelic ketamine analog, has failed to reliably recapitulate the rapid, potent anti-depressive effects of ketamine in phase II clinical studies for MDD (Iadarola et al., 2015; Sanacora et al., 2017). This raises questions about the limitations of animal models in capturing the experiential and neurobiological complexity of behavioral disorders in humans, and thus, whether demonstration of efficacy in animal models are predictive of clinical utility in humans.

The development of non-psychedelic, or non-subjective, analogs of psychedelic compounds primarily aims to benefit clinical populations for whom psychedelics’ subjective effects may be deterimental, such as those with schizophrenia or bipolar disorder (Cheung et al., 2023). Although, there is also an interest in developing these non-subjective analogues as preferable (e.g., first-inline) treatments for conditions like MDD and PTSD (Vargas et al., 2021). These compounds are also being proposed as having better risk/benefit profiles relative to traditional psychedelics, since it could in theory be impossible to have a “bad trip” or psychologically overwhelming experiences. However, a survey study of challenging experiences (i.e., “bad trips”) after psilocybin consumption found that degree of difficulty was positively associated with enduring increases in wellbeing, and 84% of participants reported that they benefited from the challenging portions of their psychedelic experience (Barrett et al., 2017). Moreover, non-subjective analogues may introduce unique risks; it is unknown how an individual’s psychology will be impacted from being in a highly neuroplastic state without corresponding changes in conscious experience to signal this alteration (Cheung et al., 2023). It is possible that the subjective experience is psychologically protective in this sense. Non-subjective psychedelics, which do not induce these beneficial psychological experiences, may not match the efficacy of subjective psychedelics in terms of effect magnitude or the duration of symptom relief. Ultimately, further research is necessary to elucidate the clinical efficacy of these novel non-subjective compounds. Studies that examine the efficacy of sub-hallucinogenic doses will also be useful in understanding the contributions of the subjective experience to clinical outcomes.

### 3.1 Network disruption and increased entropy

**FIGURE 2:**
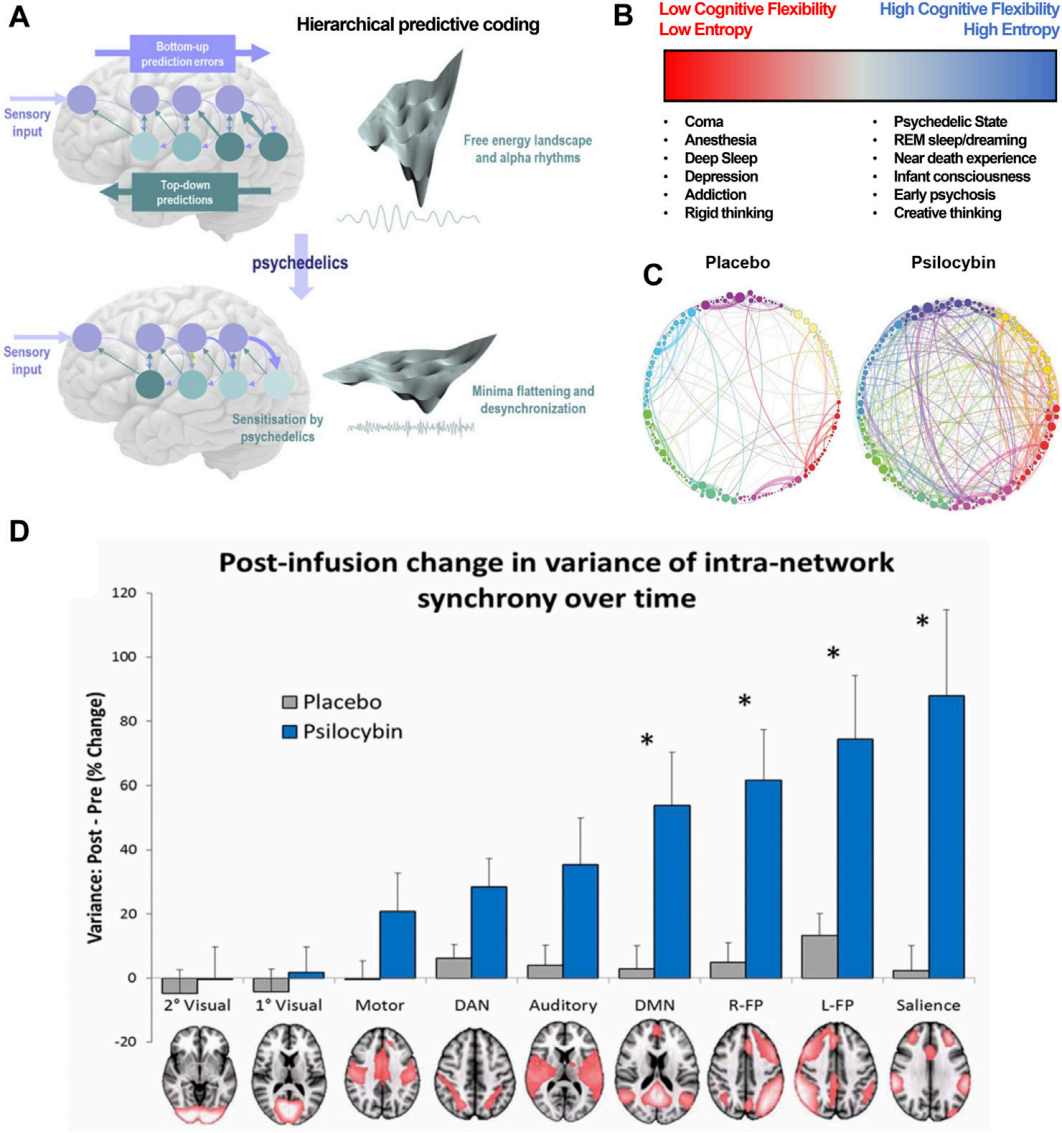
Psychedelics functionally dissolve high-level networks, disrupt hierarchical predictive coding, and induce a high entropy state. **(A)** This is a visualization of hierarchical predictive coding as used in formulating the Relaxed Brain Under Psychedelics Theory (ReBUS). In this computation architecture, sensory input arrives at the sensory epithelia and is compared with descending predictions. The prediction error is passed forward into hierarchies to update expectations at higher levels (blue arrow). Posterior expectations then generate predictions of representations in lower levels via descending predictions (teal arrow). Neuronal network computation tries to minimize the prediction errors at each level of the hierarchy, which appears as free energy minimization in the landscape of neuronal dynamics. Psychedelics sensitizes higher-level expectations to incoming sensory information and attenuates predictive coding. This can be represented as a flattening the free energy landscape, which allows computational flexibility. **(B)** This decrease in intra-network and increase in inter-network activity can also be denoted as an increase in entropy, or number of possible states within the multi-dimensional matrix of possible network states. High entropy states lend themselves to higher cognitive flexibility. **(C)** Between-network connectivity between nodes (spheres) within these networks (distinct colors) is enhanced under psychedelics, further disrupting typical function of the affected networks. **(D)** The intra-network (or within-network) variance for various neural networks, notably the DMN and salience networks, is significantly increased under the influence of psilocybin. This disruption of coordinated within-network activity is akin to functional disintegration of neural networks. **(A,D)** are adapted from Carhart-Harris *et al Pharmacol. Rev.* (2019) and Carhart-Harris *et al Front. Hum Neuroscience* (2014), respectively. **(D)** is adapted from Petri et al J. *R. Soc. Interface* (2014).

At the highest level of abstraction, psychedelics appear to have a characteristic neurobiological footprint (Figure 2A). The functional disruption of networks is suspected to (directly or indirectly) facilitate the unique psychological and phenomenological aspects of the psychedelic experience (Figure 2B). Whole-brain networks are functionally *disintegrated* and *desegregated,* i.e., decreased short-distance within-network and increased long-distance between-network connections (Figure 2C) (Carhart-Harris et al., 2014b; Müller et al., 2018).

For example, modulation of the default mode network (DMN) associated with metacognition, mental time travel, and generation of an embodied sense of self, is hypothesized to underlie the experience of drug-induced ego death (DIED) (Figure 2D) (Swanson, 2018). Interestingly, potentiated functional connectivity in the DMN is believed to underly depressive rumination and schizophrenic delusions. Furthermore, psilocybin has been shown to disrupt the anti-correlation between the DMN and the task-positive network (TPN), which is thought to maintain distinction between internal and external experience. Decreased functional connectivity between the DMN and medial temporal lobes (MTL) is observed as well during psychedelic experiences. Interestingly, MTL electrostimulation is sufficient to induce dreamlike, depersonalized experiences, similar to a psychedelic experience (Müller et al., 2018). Alternatively, the kappa-opioid receptor (KOR) dense claustrum facilitates high-order integration of input from multiple cortical areas (e.g., somatosensory, vestibular, visual), and its disruption might underly the DIED experience reported by Salvinorin A users (Stiefel et al., 2014). DIED is a cardinal component of the *mystical experience*, and *Griffiths et al* posits that the psilocybin-induced mystical experience directly mediates reductions in therapeutic outcomes in patients with depression and anxiety (Griffiths et al., 2016). These experiences can also mediate emotional breakthroughs, which have are associated with improvement of psychological wellbeing (Roseman et al., 2019).

It is worth noting that there are inconsistencies in the altered functional connectivity between specific RSNs, so it is unlikely that a single set of observed effects can serve as a neuronal correlate of the psychedelic experience. Moreover, while these disruptions of network connectivity appear to mediate the typical psychedelic experience, it is still unclear to which extent these disruptions, as opposed to individual psychological experience, facilitate the alleviation of psychological suffering (Olson, 2021; Yaden and Griffiths, 2021). Some proposals to examine this include the administration of psychedelics to unconscious individuals or the utilization of non-hallucinogenic psychedelic analogs as a means to further examine this.

Disruption of coordinated, hierarchical activity across networks causes an increase in entropy, i.e., the uncertainty and number of possible states within a system. Free energy principle posits that high-level multi-modal systems attempt to minimize free energy (e.g., uncertainty) as they integrate sensory information from lower-level unimodal systems, in such a way that the influence of lower-level systems is constrained and limited. Essentially, in order to minimize computational loads, higher-level brain networks use predictive processing, constrained by computational biases, e.g., psychological *priors*, to constantly generate and update mental models of the world based at various spatial and temporal scales (Carhart-Harris et al., 2014b; Ho et al., 2020). Predictive coding accounts for the wide range of psychological and behavioral phenomena, ranging from perceptual illusions to psychopathological states. Psychedelics, by functionally disrupting higher-level systems, potentiate bottom-up signaling and attenuate the influence of high-level priors and predictive processing which allows for greater cognitive flexibility (Figures 2A, B). Notably, networks that facilitate top-down sensory gating and integration of stimuli are also disrupted after acute or chronic psychological stress, leading to development of maladaptive priors (Carhart-Harris and Friston, 2019). For instance, patients with depression and anxiety experience enhanced negative biases in perception that are often irrational and immune to logical self-revision.

The relaxed brain under psychedelics (ReBUS) hypothesis posits that psychedelic-induced relaxation of top-bottom regulation perception facilitates an opportunity for correction of maladaptive cognitive biases that fundamentally underly psychological disorders ranging from depression to anorexia (Carhart-Harris and Friston, 2019). This proposed mechanism may account for the increases in divergent and convergent creativity following psychedelic treatment.

### 3.2 Neuronal plasticity

From a cellular-level, we can appreciate that these substances are potent neuroplasticity-inducing compounds, i.e., *psychoplastogens* (Olson, 2018). Neuroplasticity is a neuron’s capacity to reorganize its synaptic weights and connectivity within a network; it can manifest as increased neuritogenesis, spinogenesis, synaptogenesis, or neurogenesis; changes to receptor surface trafficking, conformation, or channel properties; altered vesicular neurotransmitter content, quantity, or release kinetics; or other mechanisms that involve non-synaptic factors and non-neuronal cells like astrocytes. Neuronal plasticity is the primary mediator for learning and memory, and in disease states, impaired plasticity limits the ability of the brain to adapt even when optimal conditions, e.g., non-traumatic and positive contexts, are restored. In fact, dysfunction of mechanisms regulating plasticity in key brain regions, e.g., the PFC, may underly the pathophysiology of PTSD and depression (Duman, 2004; Krystal et al., 2017).

**FIGURE 3:**
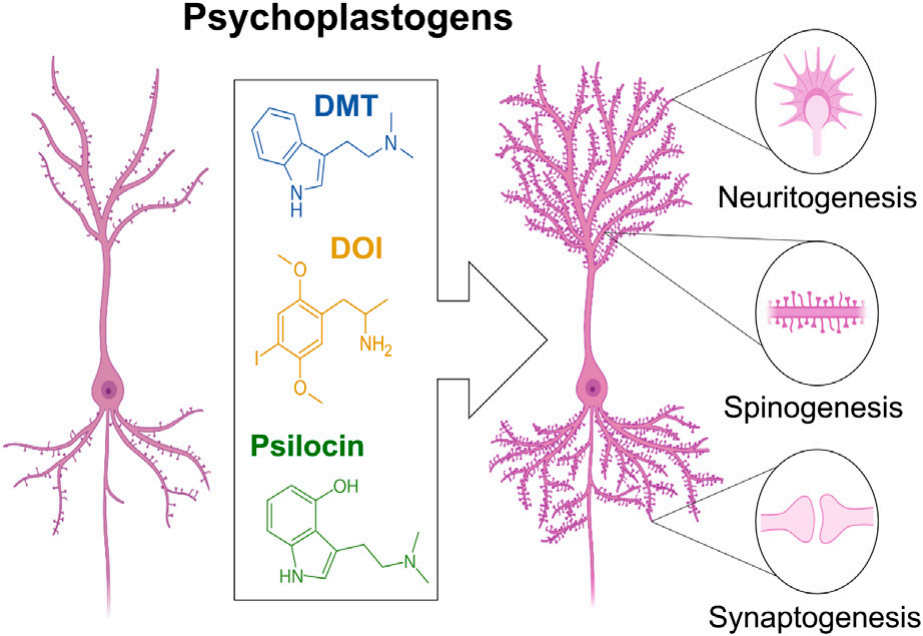
Psychedelics (e.g., DMT, DOI, LSD) are potent plasticity inducing compounds, e.g., psychoplastogens. Psychedelics can increase neuritogenesis, spinogenesis, and synaptogenesis. The capacity for psychedelics to induce neuronal plasticity might mediate therapeutic efficacy against mood disorders such as depression.

Identification of manipulations that reopen the critical period of plasticity (seen in young children) has been a priority for translational neuroscience. Recently, research showed that serotonergic phenethylamines, e.g., MDMA and DOI; tryptamines, e.g., DMT and psilocybin; and lysergamides, e.g., LSD, were able to induce 5HT_2A_-R mediated neuritogenesis, spinogenesis, and synaptogenesis in rat cortical neurons *in vitro* (Figure 3A) (Ly et al., 2018). It was also shown that noribogaine, the metabolite of ibogaine (the psychoactive compound in *T. iboga*), is a potent psychoplastogen. Moreover, a single administration of ibogain or noribogaine has been shown to induce an anti-depressive effect by increasing BDNF mRNA levels (Rodríguez et al., 2020). Importantly, serotonergic psychedelic and ketamine-induced neuroplasticity is completely blocked by antagonists of TrkB, a high affinity BDNF receptor, or mTOR, a protein kinase that regulates cell growth. Interestingly, DMT-induced plasticity is partially and fully attenuated by TrkB and mTOR antagonists, respectively, suggesting the possibility of additional BDNF-independent plasticity mechanisms (Ly et al., 2018). Furthermore, despite compounds like DMT and ketamine having similar efficacy as psychoplastogens, only DMT provides long lasting anti-depressive effects (Hibicke et al., 2020). Neurotrophic factor signaling pathways underly, at least in part, the psychoplastogenic properties of psychedelics. Additionally, there’s evidence that a psychedelic’s lipophilicity correlates with its psychoplastogenicity, with high lipophilicity enabling these compounds to traverse the cell membrane and interact with intracellular 5-HT2A receptors (Vargas et al., 2023). Additionally, these studies have revealed that the majority of cortical 5-HT2A receptors are intracellular, leading to a provocative hypothesis: serotonin might not be the endogenous ligand for the 5-HT2A receptor (Vargas et al., 2023). By employing electroporation to bolster the intracellular transport of serotonin–which ordinarily struggles to cross the cellular membrane–researchers have observed enhancements in psychedelic-like plasticity and anti-depressive effects (Vargas et al., 2023). Altogether, this implies a complex interplay of various intracellular signaling pathways that likely drive the psychoplastogenic effects of psychedelics.

Importantly, psychedelics impart distinct effects within different brain regions, but the functional significance of region-specific changes remains understudied. For instance, a single dose of by MDMA induces oxytocin-dependent plasticity in the nucleus accumbens (NA) by causing an efflux of 5-HT, indirect 5-HT4 receptor activation on oxytocin releasing neurons, and subsequent stimulation of oxytocin-terminals in the NA. This appears to reopen a critical period for social reward learning in adult mice (Nardou et al., 2019). Optogenetic stimulation of oxytocin-releasing neurons was sufficient to recapitulate these pro-social effects. Nardou et al. (2023) later demonstrated that multiple psychedelics (e.g., ketamine, ibogaine, LSD, psilocybin) not just MDMA as originally hypothesized, were capable of opening a social critical period by triggering metaplastic restoration of oxytocin-mediated long-term depression in the NA. This is one possible unifying theory by how psychedelics demonstrate transdiagnostic therapeutic efficacy. Dölen et al. hypothesized that these pro-social effects underly improvements in socialization in adults with autism and social anxiety following MDMA-assisted therapy (Danforth et al., 2018). Notably other potent plasticity promoting compounds, like cocaine, failed to reopen the social reward learning critical period, lending possible credence to the hypothesis that social reward learning in adulthood is mediating mechanism for the therapeutic efficacy of MDMA and other psychedelics (Nardou et al., 2023). While Nardou et al. (2023) found that various doses of LSD, 1–50 ug/kg, produced these effects on social critical learning period, earlier studies found that only repeated, but not acute, LSD administration (30 ug/kg, once a day, for 7 days) was able to affect social behavior in the direct social interaction experimental paradigm (de Gregorio et al., 2021). So, similar treatment protocols may produce differing modulations on immediate social behavior and (memory-related process of) social learning. Furthermore, de Gregorio et al. (2021) demonstrated that the effects of LSD on social behavior were mediated by the potentiation of mPFC excitatory transmission via 5HT_2A_/AMPA receptor and mTOR (which is also implicated in the plasticity pathways) signaling. A later study by De Gregoria et al further elucidated mechanisms underlying the behavioral effects of LSD by showing that increases in mPFC activity might also be driven by LSD-mediated attenuation of neuronal activity in the dorsal raphe nucleus activity via the desensitization of their 5-HT_1A_ receptors (De Gregorio et al., 2022). Moreover, understanding the effects of different psychedelics across distinct brain regions will facilitate an improved understanding of the modes of action, as well as the nature of the disorders.

Neuronal plasticity is crucial in memory formation, such as during the encoding phase, where the hippocampus integrates various sensory inputs into distinct neural patterns, and during the consolidation phase, where these memories transition to cortical networks. In rodents, LSD treatment during the consolidation stage reveals a bell-shaped response curve for novel object recognition, a task hippocampal dependent memory process (Orn et al., 2022). Though these researchers attribute this to decreased plasticity at higher doses, evidence indicates that LSD maintains its plasticity-enhancing effects even at these higher concentrations (Ly et al., 2018; Orn et al., 2022). This same study used *in silico* simulations of cortical-hippocampal networks (with standard memory and decision-making modules), which predicted dose-dependent memory consolidation improvements matching the experimental outcomes seen in rodents (Orn et al., 2022). This computational model interestingly implies that the prefrontal cortex’s plasticity is not essential for these effects on memory. Human studies have also demonstrated how the administration of low sub-perceptual doses of LSD (50 μg) humans, immediately after encoding is able to enhance certain types of memory consolidation (Orn et al., 2022; Wießner et al., 2022). Recent reanalysis study by Doss et al. (2022) shows that psychedelic administration during the encoding phase (which is a hippocampal memory process) impairs memory recollection. However, psychedelics appear to enhance familiarity (which is a cortical memory process) when administered during the encoding phase, potentially indicating a positive modulation of cortical memory processes. Ultimately, the effects of psychedelics on memory vis-à-vis plasticity modulation requires further investigation and may help inform the potential utility of psychedelics in specific clinical contexts.

Additional changes in neuronal morphology and function may involve changes in gene expression across different brain regions and temporal scales. For example, acute treatment with serotonergic psychedelic 5-MeO-DMT in human induced pluripotent stem cell-derived (hiPSC) cerebral organoids, revealed a modulatory effect on over 900 genes associated with long-term potentiation, dendritic neurogenesis, microtubule dynamics, and cytoskeletal reorganization (Dakic et al., 2017). Expression of various cell surface and extracellular proteins involved in regulating synaptic architecture, e.g., plexins and integrins, was also upregulated by 5-MeO-DMT. Similarly, in rodents and neuronal organoids, LSD has been shown to increase expression of genes involved in modulating synaptic plasticity, axon guidance, glutamatergic signaling, and cytoskeletal architecture (Nichols and Sanders-Bush, 2002; Orn et al., 2022). Many genes and proteins regulating plasticity are implicated in disorders like depression; There is accumulating pre-clinical evidence suggesting that psychedelic treatments may exert their antidepressant effects, at least in part, by modulating the expression of these genes and proteins (Inserra et al., 2022). The induction of plasticity by psychedelic may also underly reported increases in cognitive flexibility and creativity (Prochazkova et al., 2018; Girn et al., 2020). However, the functional and behavioral significance or causality of changes in gene expression induced by psychedelics has yet to be thoroughly investigated *in vivo*.

The contribution of plasticity mechanisms to fast-acting *versus* long-term effects of psychedelics at a cellular and network level remains a significant gap in knowledge. Likewise, contribution of elevated plasticity within a single region, or synergistic interactions across regions, on symptom improvement remains poorly understood. While psychoplastogenic properties of psychedelics are undoubtedly important in its effects and merit further investigation, we want to highlight that plasticity enhancement is not a satisfactorily defining feature of psychedelic compounds. Cocaine is also capable of triggering multiple plasticity mechanisms, i.e., dendritic arborization and synapse formation, despite having none of the psychedelic’s effects on consciousness or therapeutic utility (Lee and Dong, 2011). In fact, there is evidence that all psychedelics (e.g., ketamine, LSD, psilocybin, and ibogaine), not just MDMA, are capable of opening a *critical period* which may allow for revision of maladaptive behaviors underlying psychiatric disorders. This is likely a more distinct characteristic of psychedelic compounds and helps how psychedelics enhance standard therapeutic protocol efficacy. Nonetheless, the capacity for psychedelics to modulate a myriad plasticity regulating mechanisms implicates psychedelics as potential pharmacotherapies in diseases such as neurodegeneration and neurotrauma disorders. At the moment, there are research institutions and companies seeking to exploit the psychoplastogenic properties of psychedelics in the treatment of these classes of disorders.

### 3.3 Neuroendocrine modulation

**FIGURE 4:**
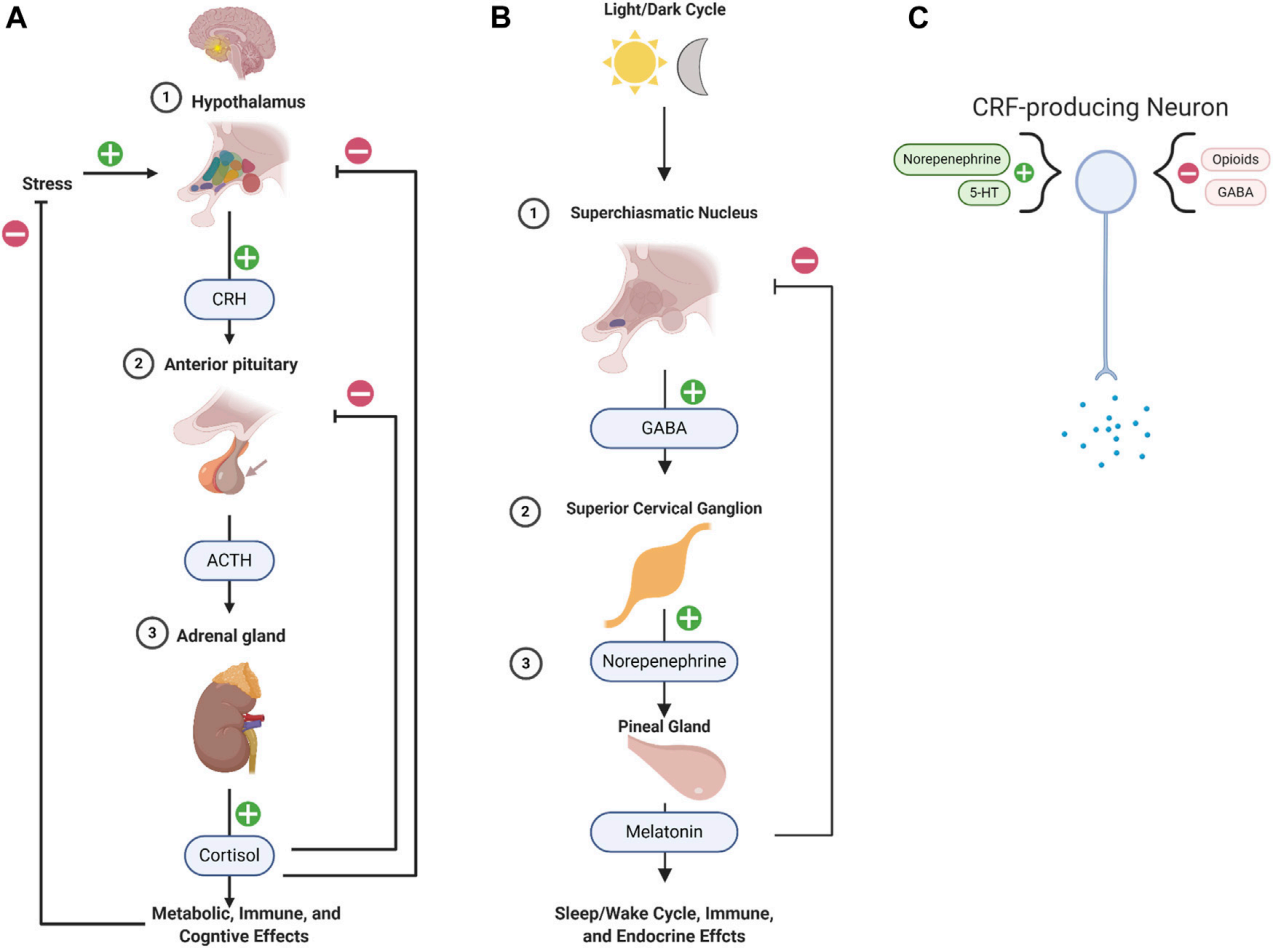
Psychedelics may modulate HPA axis signaling. **(A)** This schematic shows the healthy stress response within the Hypothalamus-pituitary-adrenal axis within the neuroendocrine system. The hypothalamus (1), which is composed of multiple sub-regions (colored), secretes corticotropin-releasing hormone (CRH), which induces Adrenocorticotropic hormone (ACTH) secretion from the anterior pituitary (2), which induces cortisol release from the adrenal gland. Cortisol imparts various metabolic effects and regulates the HPA axis in a negative feedback loop. **(B)** This schematic shows the pathway for stimulation of melatonin. In response to day-light, the superchiasmatic nucleus (SCN), a subregion of the hypothalamus, will release GABA which will allow for downstream excitation of the Superior Cervical Ganglion (SCG). The SCG will release norepinephrine directly onto the pineal gland which produces melatonin. Melatonin will regulate SCN activity in a negative feedback loop and regulate circadian rhythm, immune function, and the endocrine system. **(C)** The neurons in the hypothalamus which produce CRF can be directly excited by norepinephrine or serotonin and inhibited by opioids and GABA. Psychedelics, e.g., LSD and salvinorin A, may directly regulate HPA activity by acting directly on these CRF-producing neurons. This is merely one example, there are multiple nodes within the HPA, and other endocrine systems, which express receptors that would have an affinity to various psychedelic compounds.

In the hypothalamus-pituitary-adrenal (HPA) axis, secretion of corticotropin-release factor (CRF) from the hypothalamus induces secretory activity from the pituitary, which then induces the adrenal gland to release hormones such as cortisol and adrenaline (Figure 4A). The HPA axis is a key regulator of physiological stress, metabolism, and inflammation; cortisol has even been shown to regulate behavioral adaptation in stressful situations. Yet, its disruption, even acutely, can have long-lasting effects. For instance, exposure to traumatic and stressful events can result in HPA hyper-reactivity, immune activation, and pro-inflammatory cytokine release. Pathological HPA axis activation can lead to circadian disruption, weight gain, a chronic pro-inflammatory state (Johnson et al., 2019; Maydych, 2019). Further, HPA axis abnormalities, such as elevated or depressed baseline cortisol levels, are present in individuals with depression and PTSD, respectively (Daskalakis et al., 2013). Also, serum melatonin levels and melatonin receptor 1 immunoreactivity in key brain regions are reduced in patients with depression, and antidepressants appear to reverse this (Figure 4B) (Wu et al., 2013). In fact, melatonin might serve as a readout of noradrenergic function following treatment with antidepressants.

It is possible that psychedelics may positively regulate endocrine function via 5-HT, dopamine, or sigma-1 receptor (Sig-1R) signaling in the HPA axis (Van De Kar et al., 2001; Johnson et al., 2019). For instance, psychedelics could modulate CRF production in the hypothalamus by stimulating 5-HT receptors, and thus regulate downstream HPA axis function (Figure 4C). Moreover, MDMA and DOI can directly modulate activity in the raphe nucleus which directly regulates HPA activity via projections to the suprachiasmatic nucleus and pineal gland (Figure 4B). In fact, mescaline, DOI, and LSD have all been shown to stimulate melatonin release from rat pineal tissue *in vitro* (Shein et al., 1971; Steardo et al., 2000). LSD’s concentration is also the highest in the pituitary and pineal glands, up to 7–8 times as high as in the PFC (Passie et al., 2008).

In clinical trial participants, DOI has been shown to increase serum levels of oxytocin, prolactin, corticosterone, and ACTH (Van De Kar et al., 2001). Likewise, MDMA and MDA have been shown to increase serum levels of prolactin and cortisol in patients (Baggott et al., 2019). Another clinical study demonstrated that closely spaced repeat administration of DMT could produce a tolerance to ACTH, cortisol, and prolactin stimulation (Strassman et al., 1996). The capacity for ayahuasca, to modulate sleep parameters, e.g., REM and slow-wave sleep phases, further suggests effects on the neuroendocrine system (Figure 4B) (Barbanoj et al., 2008). Yet, the primary mechanisms driving changes in neuroendocrine function, such as indirect serotonergic action on the raphe nucleus or direct action on the hypothalamus, are unclear.

Although psychedelic modulation of the endocrine system has been shown in clinical trials and *in vitro,* there have been no assessments of changes in non-healthy volunteers, i.e., patients with PTSD or MDD, so the clinical relevance of these effects is unknown. Yet, modulation of the neuroendocrine system could measurably explain the clinical effects in patients. Ultimately, given that manipulation of the HPA axis can have demonstrable long-term effects, and that psychedelics have been shown to modulate HPA axis activity, it should be investigated as one of the therapeutic targets of psychedelic compounds (Sackler et al., 1966).

### 3.4 Immunomodulation

The theory of inflammation underlying psychiatric illness has gained traction in recent years. In fact, there is evidence that immune mechanisms, such as microglial activation, contribute significantly to pathologies underlying neurological disorders such as schizophrenia, depression, and Alzheimer’s disease (Müller, 2018; Nirzhor et al., 2018; Lee and Giuliani, 2019). There is also evidence that PTSD is associated with the presence of a low-grade inflammatory state and a significant difference in amounts of specific pro-inflammatory cytokines, acute-phase proteins, and immune cells (Speer et al., 2018). Similarly, there is an increase in pro-inflammatory immune markers, and a reduction in anti-inflammatory IL-4, in individuals with depression (Osimo et al., 2020). The significance of this is corroborated by evidence that anti-inflammatory drugs, e.g., cytokine inhibitors, statins, and minocycline, can significantly attenuate depression ratings (Bai et al., 2020). So, it is possible that anti-depressive and anxiolytic effects of psychedelics could be partially mediated by immunomodulation of a pathological inflammatory state.

Expression of 5-HT_2_ receptors on immune-associated tissue, e.g., lymphoid and thymus, as well as immune cells like microglia and macrophages might mediate a psychedelic-induced immunomodulatory effect (Flanagan and Nichols, 2018). For example, LSD *in vitro* was found to significantly inhibit proliferation of B cells and secretion of cytokines IL-2, IL-4, and IL-6 (House et al., 1994). Although anti-inflammatory action appears to be ubiquitous amongst psychedelics, DOI appears to have an especially potent anti-inflammatory effect on peripheral tissue (Nau et al., 2013). At picomolar concentrations, DOI treatment can repress tumor necrosis factr alpha (TNF-alpha) induced inflammation, expression of genes encoding adhesion molecules, e.g., ICAM-1 and VCAM-1 (whose overexpression is associated pathological vascular permeability and immune cell infiltration into tissue), and pro-inflammatory IL-6 levels in rat aortic tissue (Yu et al., 2008). (Note, there has been a recent shift in the nomenclature in which TNF-alpha is referred to as TNF and TNF-beta is referred to as Lymphotoxin-alpha.) Incredibly, hours after TNF-alpha stimulation, treatment with a single administration of DOI was still able to attenuate inflammation (Carhart-Harris1 et al., 2018). This may be mediated by DOI inhibition of nuclear translocation of NF-kB, a central genetic immunomodulator. Similarly, treatment of hiPSC-derived cerebral organoids with 5-MeO-DMT downregulates NF-kB and nuclear factor of activated T-cells (NFAT) signaling pathways (Dakic et al., 2017).

The Sigma-1 receptor (Sig-1R), which is localized to mitochondria-associated endoplasmic reticulum membrane, is a major modulator inflammatory signaling and cellular stress responses (Smith and Su, 2017). Interestingly, Sig-1Ractivation produces anti-depressant effects in rodents, and various anti-depressant drugs and psychedelic compounds, e.g., DMT and 5-MeO-DMT, are potent Sig1R agonists (Robson et al., 2012; Frecska et al., 2016). Treatment of human primary monocyte-derived dendritic cells (moDCs) with DMT or 5-MeO-DMT following lipopolysacharide (LPS) stimulation, inhibited the production of proinflammatory cytokines, increased secretion of anti-inflammatory cytokines, and inhibited moDC mediated T-cell activation (Szabo et al., 2014). Similarly, ibogaine has a high affinity for the sigma-2 receptor (Sig-2R). Sig-2Rs agonists have been shown to have immunomodulatory action by attenuating expression TNF-alpha and IL-2 by inhibition of transcription factors like NFAT.

The endocannabinoid system, composed of CB1 and CB2 receptors which are mostly expressed in the brain and by immune cells, respectively, is also involved in immunoregulation. Cannabinoid drugs have been shown to induce potent anti-inflammatory effects for patients with chronic pain, skin inflammation, and intestinal inflammation (Kunos and Pacher, 2004; Barrie and Manolios, 2017; Tóth et al., 2019). Although salvinorin A is a potent KOR agonist, direct activation of CB1 receptor mediates a synergistic ultra-potent anti-inflammatory action on LPS-stimulated macrophages *in vitro* and *in vivo*, as evidenced by significant reductions in levels of macrophage-produced nitrite, TNF-alpha, IL-10, and inducible nitric oxide synthase (NOS) (Fichna et al., 2012). Notably, salvinorin A was found to have no cytotoxicity in macrophages or effects on mitochondrial respiration. Salvinorin A, in a KOR and CB1 receptor dependent manner, was also able to reduce formalin induced inflammation-sustained pain in rodents. The indirect activation of CB1 receptors by Salvinorin A may be driven by the formation of functional heterodimers between CB1 receptors and KORs, or signaling through a common G-protein. While promising for various clinical indications, at present, the short half-life and water insolubility of salvinorin A are obstacles to clinical use.

Future investigations should assess the individual and synergistic contribution of 5-HT, Sig-1/2R, and CBD-1/2 receptor stimulation to the potent anti-inflammatory effect of psychedelics. Moreover, it is necessary to understand the contribution of the anti-inflammatory effects of on the observed clinical symptom relief. Disease models will be crucial in uncovering the full therapeutic scope of psychedelics as small-molecule anti-inflammatory agents.

### 3.5 Glial modulation

**FIGURE 5:**
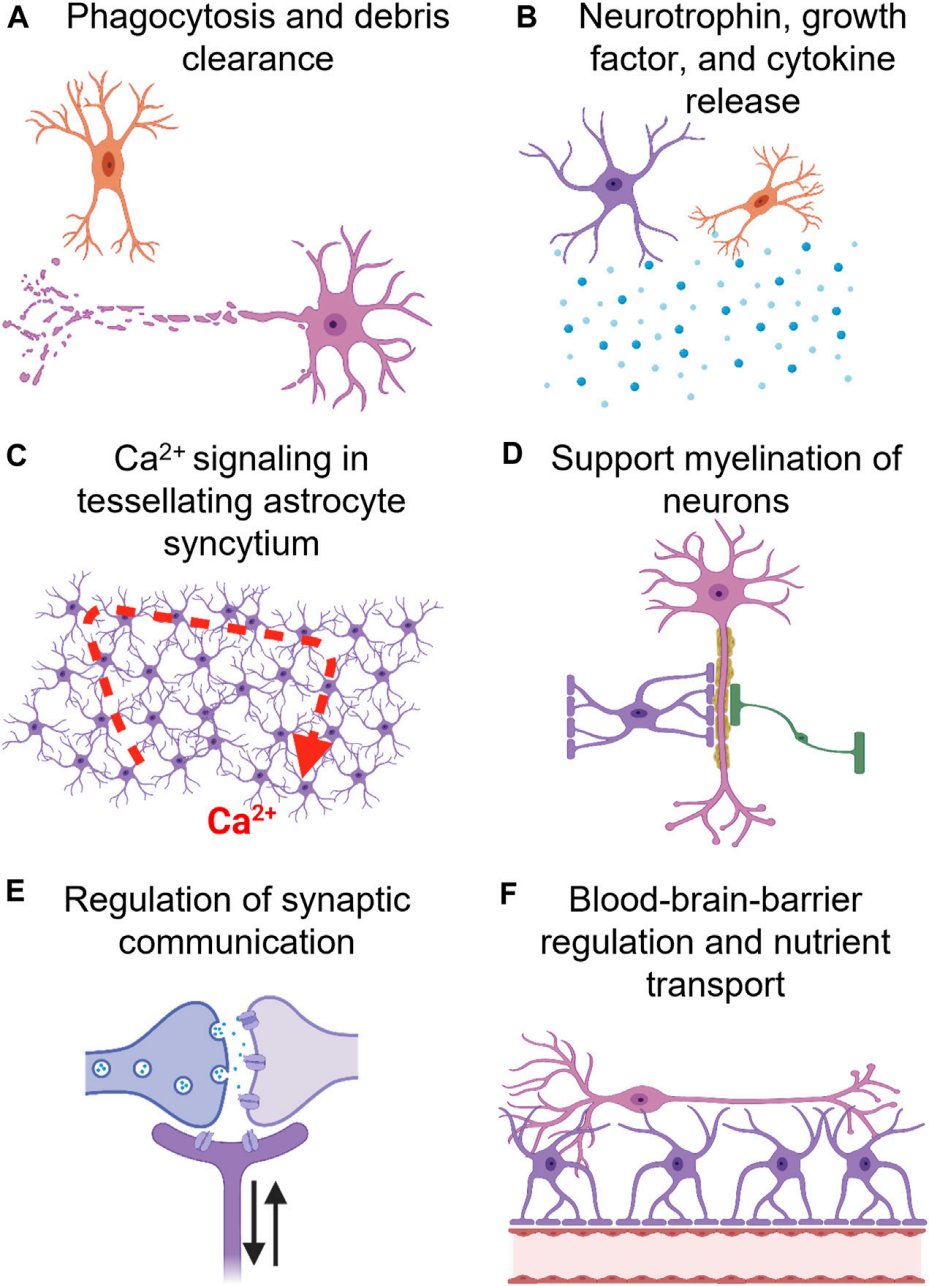
Glial cells, e.g., oligodendrocytes (green), astrocytes (purple), and microglia (orange), modulate a wide array of neuronal (orange) processes. **(A)** Microglia, the resident immune cell of the brain, mediates phagocytosis of debri, and have even been implicated in synaptic pruning (a crucial process in memory formation). **(B)** Astrocytes and microglia also release neutrophins, e.g., BDNF and GDNF, growth factors, e.g., NGF, and cytokines, e.g., IL-10. **(C)** Tessellating astrocyte networks, syncytium, composed of gap-junctions (astrocyte-astrocyte communication) and tripartite synapses (neuron-astrocyte communication) has been proposed to mediate higher-level cognitive processes. Although astrocytes are electrically inert, they mediate information processing via Ca^2+^ wave oscillations. Also, astrocytes express receptors for all neurotransmitters and neuromodulators, allowing for accurate sensing of neuronal network activity. **(D)** Oligodendrocytes primarily mediate myelination of axons, however, astrocytes regulate this process as well. **(E)** Astrocytes form a tripartite synapse by encasing neuronal pre-synaptic and post-synaptic terminals. They can regulate neuronal plasticity, e.g., synaptogenesis, and tune synaptic strength. **(F)** Astrocytes regulate blood brain barrier (BBB) activity, i.e., increase cerebrovascular blood flow in response to neuronal activity, and facilitate nutrient transport to neurons.

Glial cells are indispensable to healthy brain function. They regulate synapse formation and transmission while also providing metabolic support to neurons and their dysregulation can contribute to disease and behavioral or cognitive impairment (Figure 5). Astrocytes, specifically, are notable given that their functional complexity increases throughout our phylogenetic tree, suggesting their involvement in the genesis of human behavior and cognition (Fan and Agid, 2018). Incredibly, a single astrocyte’s capacity to form up to 140,000 tripartite synapses could also serve as a means of computationally integrating activity from a large population of neurons (Robertson, 2013; Shan et al., 2021). Further, it is possible that the complex geometric matrix constructed by seamless tessellating astrocyte domains, functionally connected by gap junctions, provides a template for the generation of consciousness via integration of whole-brain information across multiple spatial and temporal scales (Robertson, 2013).

Changes in glial function are also associated with mood disorders, e.g., depression and obsessive-compulsive disorders, neurodegenerative disorders, e.g., Parkinson’s disease, and (e.g., TBI and stroke). In traumatic brain injury (TBI), astrocytes and microglia persist in a reactive pro-inflammatory state which can negatively impact neuronal function and blood-brain barrier (BBB) permeability. In Parkinson’s disease, impairment of endogenous microglial phagocytosis may contribute to the chronic pathology of the disease. Understanding the effects psychedelics impart on glial cells might reveal an entirely new avenue for therapeutic applications.

Given that astrocytes can express virtually all known neurotransmitter receptors, and are functionally coupled to neurons, it is unfathomable that they are not affected by psychedelic compounds. Also, since psychedelics are potent psychoplastogens, it is possible that astrocyte morphology and functionality would be dramatically affected by psychedelics in response to robust structural and functional changes in the neuronal population (Ly et al., 2018). In fact, the rapid anti-depressive effects of (S)-ketamine in rats are associated with an increase in astrocyte size as well as length and number of astrocyte processes in the CA1 subregion of the hippocampus, an area crucial in learning and memory formation (Ardalan et al., 2017). Moreover, glial derived neurotrophic factor expression (GDNF) is potentiated in multiple brain regions by ibogaine and is both necessary and sufficient for mediating anti-addictive effects (Marton et al., 2019). Also, salvinorin A-induced KOR stimulation increases proliferation of astrocytes *in vitro*. Furthermore, salvinorin A mitigates microglial and astrocyte pro-inflammatory activation *in vivo* following treatment with formalin (Mclennan et al., 2008; Guida et al., 2012). DMT and psilocin have also been shown *in vitro* to modulate microglia functionality, specifically, decreasing pro-inflammatory signaling and phagocytic activity (Kozłowska et al., 2021).

It is possible that modulation of glial expressed circadian clock regulators, e.g., *PER1*, may also underlie psychedelic induced changes in sleep/wake cycles (Kay and Martin, 1978; Barbanoj et al., 2008; Gould et al., 2020). For instance, ayahuasca administration has been shown to decrease REM power, increase REM onset, and decrease REM duration, but increase slow wave sleep (SWS) power in the first night cycle. Likewise, LSD administration in felines has been shown to decrease REM, while increasing wakefulness and drowsiness. In fact, astrocyte transcription-translation negative feedback loops alone can drive circadian behavior and molecular oscillations (Brancaccio et al., 2019). Interestingly, DOI has been shown to transcriptionally activate astrocytes and increase *cfos* and *PER1* expression in the somatosensory and prefrontal cortex (Martin and Nichols, 2016; Kyzar et al., 2017). It is possible that psychedelic action on neuronal populations is directly mediated and dependent on glial function. Thus, it is necessary to understand the multi-faceted influences and related direction(s) of causality between astrocytes and neurons following psychedelic treatment.

### 3.6 Epigenetics

**FIGURE 6:**
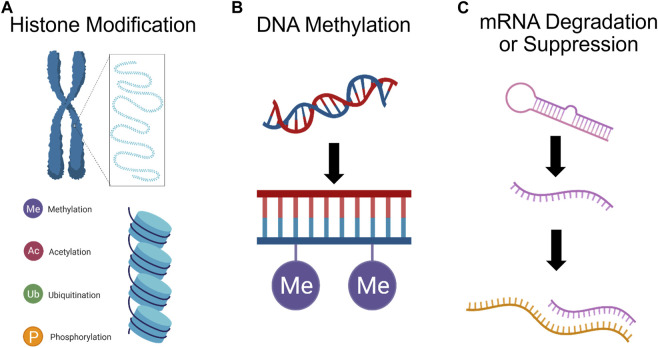
Psychedelics can affect gene expression. **(A)** Histone (blue cylinder) modification, e.g., methylation, acetylation, ubiquitination, and phosphorylation, can impact gene expression by adjusting chromatin morphology. **(B)** DNA methylation, by which methyl groups are added to the DNA backbone, can suppress gene transcription. **(C)** Finally, miRNA can cleave mRNA and repress translation, or mark mRNA for degradation., ultimately silencing expression.

Epigenetic processes, i.e., regulation of gene expression and protein translation by DNA methylation, histone modification, and mRNA degradation or suppression (Figure 6), is being considered as a potential mechanism in mediating long-lasting vulnerability to psychological and physiological disorders (Tietjen et al., 2010; Maleki et al., 2012). For instance, there is a strong association between DNA methylation of the BDNF promotor gene and PTSD diagnosis in combat-exposed veterans (Kim et al., 2017). Incorporating epigenetic mechanisms into our understanding of diseases, e.g., substance abuse and depression, may elucidate why transgenerational risk factors are associated with most mental illness (Bowers and Yehuda, 2016; Klengel et al., 2016; Burchert et al., 2017). This also helps to bridge our understanding of how non-genetic factors such as sex hormones, stress hormones, and inflammation can modulate psychological wellbeing (Smart et al., 2015).

While the capacity for psychedelics to exploit epigenetic mechanisms in order to mediate clinical symptom reduction has not been explicitly assessed, there are interesting findings which merit further study. For instance, LSD has been shown to rapidly increase histone acetylation in rabbit brain tissue (Brown and Liew, 1975; Martin and Nichols, 2016). Chronic LSD administration has also been shown to modulate DNA methylation at 635 CpG sites and cause differential expression of 178 proteins in the mouse PFC; however, only 4 genes and their protein product were identified as having greater methylation at intragenic CpG sites, increased transcription level, and greater expression. This limited overlap between DNA methylation and proteomic changes suggests that DNA methylation by psychedelics may be sufficient for driving changes in protein expression (Inserra et al., 2022). Another study assessing associations between histone modifications (H3K27ac) and their correlations with gene expression found that a single dose of DOI is sufficient for inducing changes in chromatin organization leading to increased activation of enhancers associated with synaptic assembly and function (de la Fuente Revenga et al., 2021). Interestingly, the majority of differentially expressed genes exhibited transient changes in expression levels and were not directly correlated with enhancer dynamics, indicating that immediate transcriptomic modifications play a minor role. Instead, the enduring impact of DOI on gene expression in the frontal cortex primarily arises from long-lasting (at least 7 days) epigenomic modifications. These findings suggest that the psychedelic’s lasting effects are primarily mediated through stable changes in the epigenome, rather than immediate transcriptomic alterations, which were relatively transient. Also, ketamine has been shown to induce anti-depressive effects via stimulation of histone deacetylase 5 (HDAC5) phosphorylation and nuclear export in rat hippocampal neurons (Choi et al., 2015). Furthermore, ketamine induced phosphorylation of HDAC5 has been shown to mediate upregulation of *Bdnf* promoter IV activity and expression of *Bdnf* exon IV mRNAs in the hippocampal neurons (Choi et al., 2017).

The Modern Spirit research project, in collaboration with Multidisciplinary Association for Psychedelic Science, recently published outcomes from a pilot study examining whether epigenetic changes are associated with observed reduction of patient PTSD scores. The pilot study had a small sample size, so while 37 out of 259 CpG sites identified were significantly predictive of symptom reduction, only 2 CpG sites, *CRHR1* and *NR3C1*, remained significant when subjected to false discovery rate corrections. The *CRHR1* site had the most significant change in methylation in the MDMA group relative to the placebo group. These changes in *CRHR1* and *NR3C1* agree with previous studies that assessed methylation changes following regular psychiatric treatment for patients with PTSD (Yehuda et al., 2013; Pape et al., 2018). It is possible that with a larger study, the more of the 37 CpG sites identified may be found to be significant predictors of symptom reduction (Lewis et al., 2023b).

Psychedelics, if truly potent inducers of epigenetic changes, might be an ideal tool for research into the epigenetic underpinnings of mental illness. To advance general psychiatry and develop a sound understanding of the mechanisms behind the long-term effects of psychedelics, human studies should strive to incorporate relatively low-cost epigenetic analysis.

### 3.7 Gut-brain axis

**FIGURE 7:**
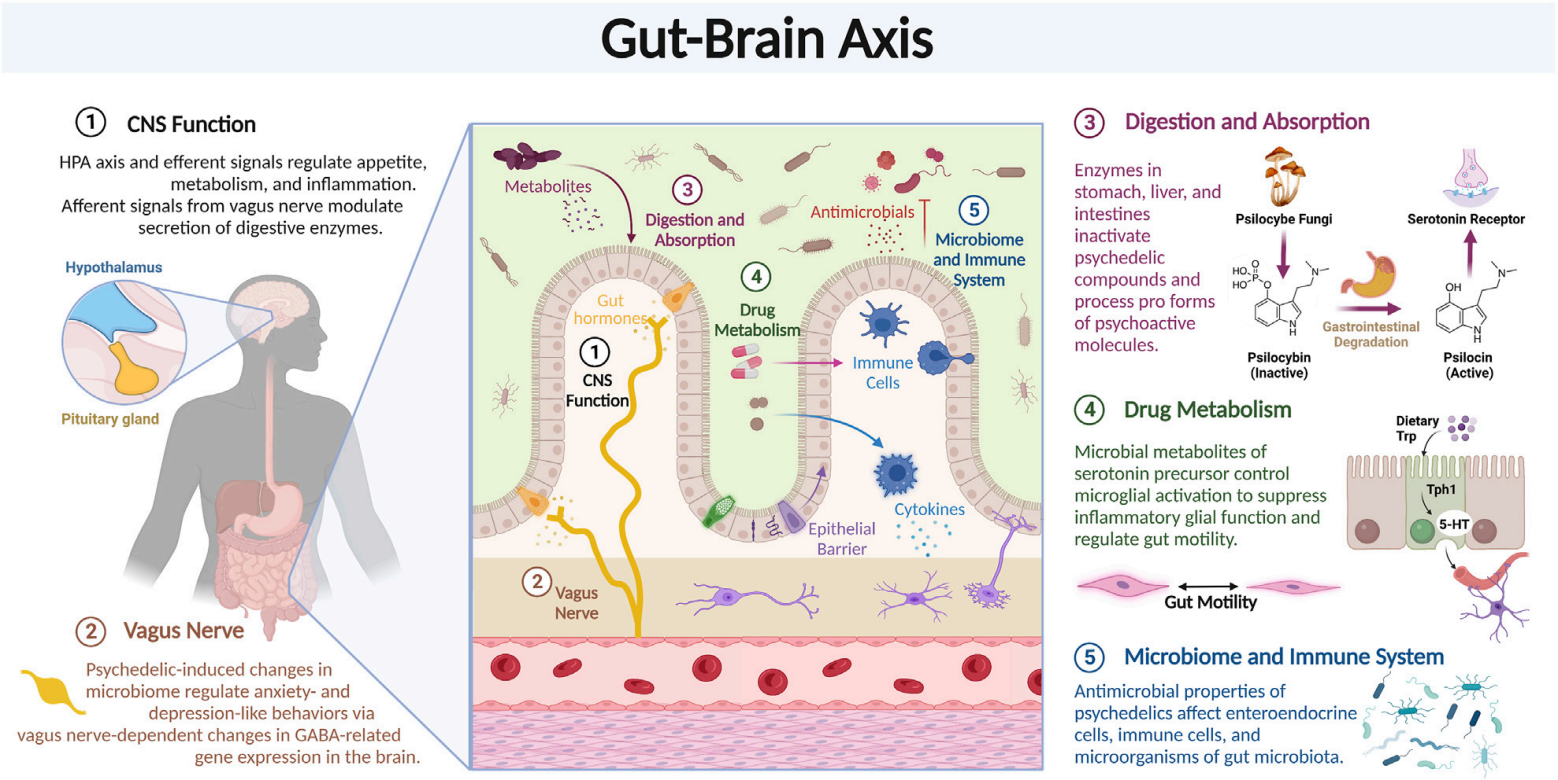
Psychedelics can affect gene expression. Review of the various components of the Gut-Brain axis which may modulate the effects of psychedelic compounds.

In addition to effects on targets in the brain, psychedelics may also act through other organs, such as the gastrointestinal (GI) tract (Figure 7). Bidirectional communication between the GI tract and the brain via “the gut-brain axis” maintains homeostasis in both organs, e.g., microglial development and immune function, blood brain barrier permeability, intestinal immune tolerance, and more (Agirman and Hsiao, 2021). In the past decade, the gut-brain axis has been increasingly implicated in the pathogenesis and treatment of anxiety, depression, substance use disorders, and numerous other psychiatric and neurodegenerative disorders. It is regulated at the neural, endocrine, and immunological levels; involving roughly 200 million enteric neurons and glial cells, specialized enteroendocrine cells, over 70% of the body’s immune cells, and trillions of microorganisms collectively known as “the gut microbiota” (Schneider et al., 2019). Notably, many of these cellular targets, including some bacteria, express the same receptors as in the brain, suggesting direct action of psychedelics on this system via the same modes of action described above (Fung et al., 2019).

While the link between gastrointestinal and behavioral symptoms is well-characterized, mechanistic causal explanations are an active area of investigation. One clinical research area involves the vagus nerve, the main arm of the autonomic nervous system, and part of the gut-brain axis, that carries neural signals to and from the digestive system, including the GI tract, and the brain. The former (efferent signals from the brain) modulate the secretion of digestive enzymes and the HPA axis and the latter (afferent signals to the brain), which make up the vast majority (>90%) of neural signals, regulate appetite, metabolism, and inflammation (Brei et al., 2018). Importantly, vagus nerve stimulation is an FDA-approved treatment option with anti-inflammatory properties and moderate efficacy for treatment-resistant depression (Daban et al., 2008).

Recent preclinical research supports the role of members of the gut microbiota in regulating brain behavior. Most psychedelics and even *Peganum harmala,* the plant containing harmine and harmine-like compounds used in Ayahuasca, have antimicrobial properties; however, scientific reports on their effects on the gut microbiota are scarce, low resolution, limited to *in vitro* tests, only test for pathogens, and/or are highly variable (Moloudizargari et al., 2013; Thompson and Szabo, 2020). One study showed that low-dose ketamine administration in male Wistar rats increased genus levels of lactic-acid producing *Lactobacillus* and serotonin-responsive *Turicibacter,* both of which are negatively associated with disorders like depression (Getachew et al., 2018). Another study showed that LSD mediated increases in pro-social behavior and modifications to hippocampal endocannabinoid signaling were accompanied by alterations to the composition and diversity of the gut microbiota. Specifically, LSD decreased gut microbial diversity and prevented the decrease in Fimicutes:Bacteroidetes ratio, as well as alterations in the abundance of Bifidobacterium, Ileibacterium, Dubosiella, and Rikenellaceae RC9 (de la Fuente Revenga et al., 2021). Research on strain-level differences could further inform how ketamine is changing microbial gut ecology; *Lactobacillus rhamnosus,* but not *Lactobacillus salivarius,* was found to regulate anxiety- and depression-like behaviors in mice, which occurred via vagus nerve-dependent changes in GABA-related gene expression in the brain (Bravo et al., 2011). Further studies should examine the causal relationship between the effects of psychedelics on the gut microbiome and neuronal circuits.

Functionally similar groups of gut microbiota can metabolize a variety of dietary components, and their byproducts can have direct effects on CNS inflammation. A recent report identified a cellular pathway in a mouse model of multiple sclerosis where microbial metabolites of tryptophan, a serotonin precursor, control microglial activation to suppress inflammatory astrocyte function (Rothhammer et al., 2018). However, considering the lack of research with psychedelics and the gut microbiota, it is difficult to formulate data-driven hypotheses regarding how they may affect microbial metabolite profiles to induce such effects on the brain. In sum, future studies should first increase genetic resolution to the strain-level and incorporate functional pathway or metabolomic analyses to ascertain the constituents and functionality of the gut microbiota as a foundation to evaluate potential psychedelic-induced changes.

Indeed, work elucidating the role of the gut-brain axis in psychedelics is in its infancy and currently limited to speculation or ongoing research. One recent hypothesis supports the idea of a more dominant role for the microbiota in microdosing psychedelics (Kuypers, 2019). In 2020, Heroic Hearts Project, a veterans group supporting psychedelic research, began collaborating with academic institutions to elucidate how Ayahuasca changes the gut microbiota of veterans with PTSD and how behavioral biomarkers correlate with these changes (Gould et al., 2020). Last year, Nova Mentis, a Canadian biotechnology company focusing on psilocybin-based therapeutics, launched a large (>300 person) initial observational study to assess microbiota differences across different subtypes of autism spectrum disorder compared to controls (Rascan, 2021). Moreover, diet and nutrition is the biggest modulator of the microbiota, and thus GI function, and should be incorporated into future pre-clinical and clinical studies (Hasty, 2022). Finally, psychedelic effects in the GI tract may have synergistic, additive, or antagonistic effects with effects on the brain and are a critical area of preclinical and clinical research that serves as an important steppingstone in understanding how these substances affect the entire body.

## 4 Novel applications for psychedelics

In the coming decade, researchers will continue to unravel modes of psychedelic action across multiple physiological systems, as well as the full therapeutic potential of these compounds. While human research is the most clinically relevant and translatable, it lends itself poorly to thorough investigation of cellular mechanisms. In fact, due to their experimental manipulability, animal models and *in vitro* models will be crucial in furthering our understanding of psychedelics. In this section, we will address novel clinical considerations for psychedelics, as well as existing tissue and animal models which lend themselves to investigating these applications.

### 4.1 Neurodegenerative disorders

**FIGURE 8:**
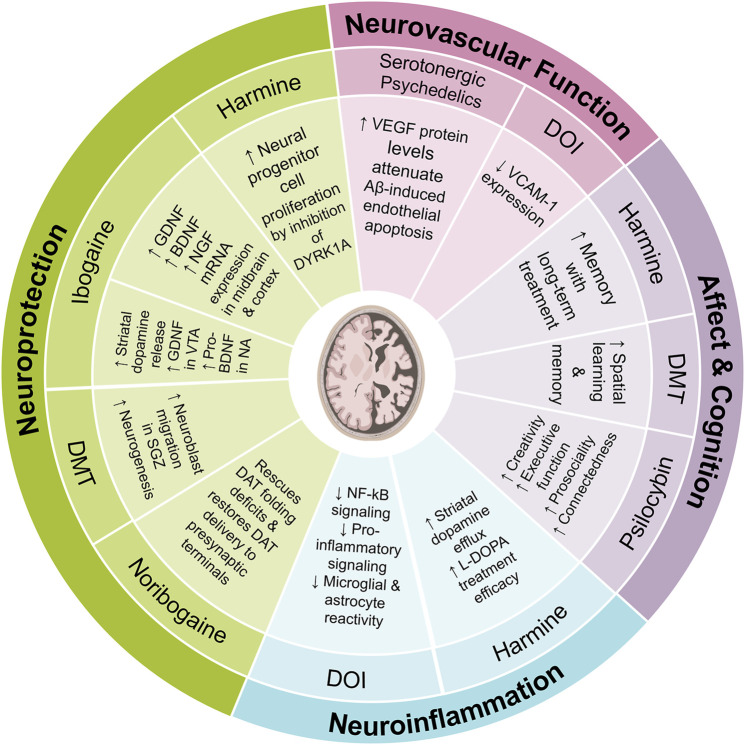
Review of possible mechanisms of action by which various psychedelic compounds may attenuate the pathophysiology of and enhance recovery from neurodegenerative disorders.

Neurodegenerative diseases are characterized by a progressive loss of neurons and subsequent impairment of motor, cognitive, and emotional faculties. Although there are behavioral (e.g., opioid use disorder), environmental (e.g., pollution), and genetic factors that predispose people to developing neurodegenerative disorders, such as Alzheimer or Parkinson disease (AD or PD), aging is the primary risk factor. Currently, therapeutics for AD or PD are only meant to relieve symptoms, rather than pausing or reversing progression of the underlying neuropathology. Various psychedelic substances, e.g., ayahuasca and ibogaine, impart neuroprotective effects in patients with neurodegenerative disorders (Samoylenko et al., 2010; Fisher et al., 2017; Katchborian-Neto et al., 2020). Overall, there is evidence that psychedelics may address multiple facets of the neuropathology of neurodegenerative disorders, such as AD and PD (Figure 8).

#### 4.1.1 Parkinson’s disease

Parkinson’s disease (PD) is characterized by accumulation of misfolded alpha-synuclein aggregates and subsequent degeneration of midbrain dopaminergic neurons in the substantia nigra (SN), which leads to progressively severe motor deficits. Administration of dopamine agonists or precursors can attenuate motor symptoms but cannot prevent the inevitable progression of the pathology. However, pre-clinical studies have found GDNF to induce sprouting of dopaminergic fibers and facilitate improvement in symptoms of Parkinson’s (Sullivan and O’Keeffe, 2016). Yet, given that GDNF is not permeable across the blood brain barrier, its clinical utility is greatly limited.

Recent research has shed light on the intriguing effects of ibogaine on the expression levels of key neurotrophic factors such as GDNF, BDNF, and NGF. For instance, a study examining the VTA, SN, PFC, and NAcc at 24 h post-treatment with ibogaine revealed increases in GDNF expression in VTA and SN, but not the PFC and NAcc, while BDNF and NGF expression increased across all brain regions (Marton et al., 2019). Interestingly, while BDNF mRNA levels increases were more pronounced (∼5-fold) compared to GDNF and NGF, this did not translate into a higher level of mature BDNF protein, a somewhat unexpected finding (Marton et al., 2019). Despite the substantial changes in growth factor expression induced by ibogaine in specific brain regions, only the changes in GDNF expression seem to lead to higher levels of mature GDNF in the VTA (Marton et al., 2019). In fact, it seems that induction of GDNF by ibogaine may activate an autocrine loop that leads to long-term synthesis and release of GDNF. Of note, due to safety concerns around ibogaine, which can cause cardiac arrythmia by impairing cardiac ion channel function, researchers have developed a non-hallucinogenic non-toxic analogue, tabernathalog (Belgers et al., 2016; Cameron et al., 2021). While tabernathalog retains the capacity to promote structural neural plasticity, reduce alcohol- and heroin-seeking behavior, and anti-depressive properties, its effects on various growth factors still needs to be assessed (Belgers et al., 2016; Cameron et al., 2021). These insights warrant further investigation to unravel the precise mechanisms and potential implications when developing ibogaine, and ibogaine-analogs, as therapeutics for PD.

PD is also characterized by mutations that cause folding defects in the dopamine transporter (DAT) which impairs healthy dopaminergic neurotransmission. Noribogaine has been shown in a *drosophila* model system to function as a pharmacochaperone and rescue folding deficits in human DAT variants associated with juvenile PD, as well as restore the delivery of DAT to the pre-synaptic terminals of dopaminergic neurons (Kasture et al., 2016; Mazhar Asjad et al., 2017). These findings have driven the development analogs that are more efficacious pharmacochaperones (Bhat et al., 2020).

Ayahuasca, a psychedelic Amazonian decoction created from harmine-containing *Banisteriopsis caapi* vines and DMT-rich *Psychotria viridis* leaves, is also a promising therapeutic for PD. Studies have shown that crude extracts of *B. caapi* alone can improve motor symptoms in PD patients (Wang et al., 2010), however, there are dozens of anti-oxidative and neurorestorative compounds that may contribute to these improvements. For example, harmine, an MAO-A inhibitor, has been shown to increase proliferation of neural progenitor cells *in vitro* by inhibition of DYRK1A, whose overexpression is associated with PD and AD pathophysiology (Dakic et al., 2016). Harmine has also been shown to induce striatal dopamine efflux *in vitro* and *in vivo* in rats, as well as improve efficacy of L-DOPA treatment in marmoset PD models (Fisher et al., 2017). However, Neto et al. demonstrated that harmine exerted smaller neuroprotective and proliferative effects, compared to ayahuasca or crude extracts of its constituent plants, in an *in vitro* model of Parkinsonian neurodegeneration (Katchborian-Neto et al., 2020). This same study found that DMT had no significant neuroprotective effects. Ultimately, this supports a holistic approach in plant medicine research. We should consider that the medicinal effects of traditional plant medicine are not attributable to a single compound, instead, various metabolites likely act synergistically or by multiple mechanisms to obtain putative pharmacological efficacy (Katchborian-Neto et al., 2020). Furthermore, there are many traditional methods for preparing the ayahuasca brew, whose biological effects and chemical composition have yet to be assessed.

There are various ways to study pathological characteristics of PD *in vitro,* however, there is no model capable of recapitulating all aspects of PD etiology. PD modelling *in vitro* can involve the use of proliferative cell lines, whose oncogenic origin and absence of neuronal properties is limiting, or primary cell cultures, which have low-reproducibility and absence of native functionality (Ferrari et al., 2020). Indeed, the most translational studies are implemented in 3D hiPSC cultures and/or midbrain organoids which are genetically modified to overexpress PD-related genes (Ferrari et al., 2020). Our group has developed a 3D tissue engineered nigrostriatal pathway (TE-NSP) capable of recapitulating the structure and function of the nigrostriatal pathway, which is central in the neuropathology of Parkinson’s (Struzyna et al., 2018; Gordián-Vélez et al., 2021; Struzyna et al., 2022). This TE-NSP consists of distinct dopaminergic and striatal neuronal aggregates embedded at opposite ends of a hydrogel micro-column, and subsequently the formation of long axonal tracts between both spheroids. By exposing the cultures to alpha-synuclein, this tool can be used to interrogate the effects of psychedelics on PD-associated synucleinopathy, axonal degeneration, and neuronal loss. We have demonstrated that organoids embedded in micro-columns will grow biomimetic long-axonal tracts, thus Parkinsonian organoids could be used produce a humanized PD testbed with long-axonal tracts (Cullen et al., 2018; Clark et al., 2020).

Animal models can complement these *in vitro* testbeds; however, most PD animal models involve acute exposure to neurotoxins such as 6-Hydroxydopamine hydrobromide (6-OHDA) or 1-methyl-4-phenyl-1,2,3,6-tetrahydropyridine (MPTP) which rapidly induce dopaminergic cell death. While neurotoxin models rapidly produce measurable motor deficits and are comparable across studies, they scarcely recapitulate the actual pathological features of PD. Instead, PD animal models that induce gradual, chronic cell loss via direct alpha-synuclein injections to the midbrain or mutations to PD-relevant genes, would be ideal for assessing whether specific psychedelics can slow or reverse pathological progression (Buhidma et al., 2020).

#### 4.1.2 Alzheimer’s disease

Alzheimer’s disease (AD) is characterized by beta-amyloid accumulation, phosphorylated tau formation, chronic hyperactivation of glial cells, and neuronal loss (Serrano-Pozo et al., 2011; Nirzhor et al., 2018). This manifests in cognitive and behavioral deficits, e.g., declines in creativity and executive functioning, as well as lower scores in openness to new experiences and empathy. Current treatments include acetyle cholinesterase inhibitors and NMDA-R antagonists, however, these only attenuate symptoms and do not modify the underlying pathology. In contrast, behavioral interventions, e.g., increased socialization, mental stimulation, and exercise are known to reduce risk for developing AD in vulnerable populations and greatly mitigate further pathological decline (Hsiao et al., 2018; Mushtaq et al., 2014; Ruthirakuhan et al., 2012).

Notably, psychedelics have been shown to positively impact (divergent and convergent) creativity and executive function, as well as increase traits of openness and empathy in healthy volunteers (Erritzoe et al., 2018; Mason et al., 2019; Aday et al., 2020). The pro-social effects of psychedelics, e.g., improvements in communication with others and improvements in relationship with friends and family, may also be beneficial for AD patients (Carhart-Harris et al., 2018). Patients in psychedelic-assisted treatments often report being able to re-connect with family members, friends, and strangers. In fact, patients identified increased connection as one of the two main change processes (Preller and Vollenweider, 2019). Psilocybin treatment has also been shown to increase extraversion and openness (Erritzoe et al., 2018). Furthermore, the safety and tolerability of psychedelics, specifically LSD, has been demonstrated in populations of volunteers aged 55–75 years of age (Family et al., 2020). The potential for psychedelics as a novel AD treatment is not missed; in fact, Johns Hopkins is currently investigating whether psilocybin can help attenuate and/or reverse cognitive impairment in early AD.

Nonetheless, impaired or attenuated neuronal plasticity is a major contributor to age- and AD-related cognitive deficits, and *in vivo* studies have shown a reversal of deficits upon potentiation of neuronal plasticity. Given the significance of compensatory brain mechanisms, e.g., neural plasticity, in improving cognitive function in early-stage AD, it is possible psychedelic psychoplastogens will provide therapeutic benefits (Hill et al., 2011). Interestingly, in adult rodents DMT has been shown to promote neurogenesis and neuroblast migration in the subgranualar zone of the dentate gyrus, which highly expresses Sig-1Rs, and subsequently improve spatial learning and memory (Morales-Garcia et al., 2020). It is worth noting that blocking Sig-1Rs blocked these effects on neurogenesis. However, using a Aβ1–42 injected mouse model of AD, it was observed that DMT had a negative impact on neurogenesis, and in contrast PRE084, a highly selective Sig-1R agonist, was found to enhance the process of neurogenesis (Borbély et al., 2022). DMT’s 5-HT_2A_ receptor agonism, through an unknown process, mediates neurogenesis impairment in AD-like, but not healthy, brains. Thus, it is uncertain whether psychedelic induced neural plasticity will generate appreciable cognitive improvement in patients experiencing cognitive decline.

There is an abundance of rodent behavioral paradigms to assess executive function, learning and memory, as well as creativity paradigms which would be useful in exploring the potential for psychedelics in AD. Given that most models of AD tend to only recapitulate part of the neuropathology, it would be worth assessing these outcome measures in aged rodents. Attenuation of age-related cognitive impairments could also be examined against accumulation of amyloid beta protein and changes in synaptic plasticity, while also leveraging an array of transgenic rodent models demonstrating multi-faceted features of PD pathology.

While the potential cognition enhancing, behavioral, and pro-social effects of psychedelics merit consideration, the immunomodulatory capacity of psychedelics suggests the potential for treating aspects of the underlying neuropathology of AD. For example, psychedelics such as DOI have been shown to attenuate NF-kB signaling which drives proinflammatory signaling, including microglial and astrocyte reactivity, that contributes to AD pathology (Szabo, 2015). However, in a Aβ1–42 treated mouse model of AD, DMT was effective at attenuating astrocyte, but not microglia, reactivity (Borbély et al., 2022). Long-term harmine administration has also been shown to improve memory in mouse models of AD, but it is unclear whether this is mediated by antioxidant action, anti-inflammatory action, or enhancement of cholinergic transmission (Li et al., 2018). Unfortunately, the majority of anti-inflammatory effects of psychedelics have been mostly assessed against TNF-alpha or LPS induced inflammation, so the therapeutic efficacy against the complex range of signaling pathways that could trigger pro-inflammatory and neurodegenerative states is currently understudied.

Just as with PD, *in vitro* test beds capable of recapitulating the pathological hallmarks of AD, e.g., Aβ aggregation and neuroinflammation, will be powerful tools for early research into psychedelics and early AD. For instance, Park et al developed a 3D tri-culture model in which human-derived neurons are transduced to overexpress Aβ precursor protein (APP) in order to induce AD-like pathophysiology (Park et al., 2018). Furthermore, this model is unique because it permits investigation of microglial recruitment, glial reactivity, and neuroinflammation in AD (Rothhammer et al., 2018). Although the capacity of psychedelics to attenuate inflammation in peripheral tissue is well understood, it remains to be seen whether psychedelics can reverse or prevent glial changes into a pro-inflammatory phenotype in the brain.

Another facet of AD pathophysiology, which deserves attention as a possible therapeutic target is the relationship between microvascular dysfunction and amyloid-beta accumulation (Joo et al., 2017). Specifically, in AD there are changes in regulation of blood flow, vessel integrity, and fluid dynamics, however it is possible that cerebrovascular dysfunction precedes cognitive decline and neurodegenerative changes. Psychedelics might improve vascular functions by multiple complementary mechanisms, such as increasing protein levels of vascular endothelia growth factor (VEGF) which has been shown to attenuate Aβ -induced endothelial apoptosis *in vitro* (Furrer et al., 2011). DOI has also been shown to reduce vascular cell adhesion protein 1 (VCAM-1) expression, which is elevated in AD patients (Yu et al., 2008; Nau et al., 2013).

There are robust *in vivo* models for assessing whether psychedelic modulation of microvasculature function can mediate improvements in AD pathology. Held et al. (2017) demonstrated that hypertensive stroke-prone rats, a model of cerebral small vessel disease, had higher amounts of amyloid-beta build up in small blood vessels within the cortex, striatum, and hippocampus. Further, a transgenic (TGF344) AD rat model, which has been shown to display neurovascular network dysfunction at an early stage of AD-like pathology, is also well suited to this investigation (Joo et al., 2017). These models would aid in exploring therapeutic effects of psychedelics which are mediated by modulation of neurovasculature function (Rabinowitz and Levin, 2014).

Altogether, there are multiple effects of psychedelics which prompt their consider as potential therapeutics for AD. However, given our poor understanding of the modes of action for psychedelics, and the lack of comparative *in vivo* studies, it is challenging to determine which psychedelic compound(s) could be efficacious. The aforementioned *in vitro* and *in vivo* models are great tools for AD research, specifically, because they permit cell type specific screening to test multiple mechanisms, e.g., attenuation of astrogliosis or neurovascular deterioration, by which psychedelics may slow, stop, or reverse neurodegeneration in patients with AD.

### 4.2 Brain injury disorders

**FIGURE 9:**
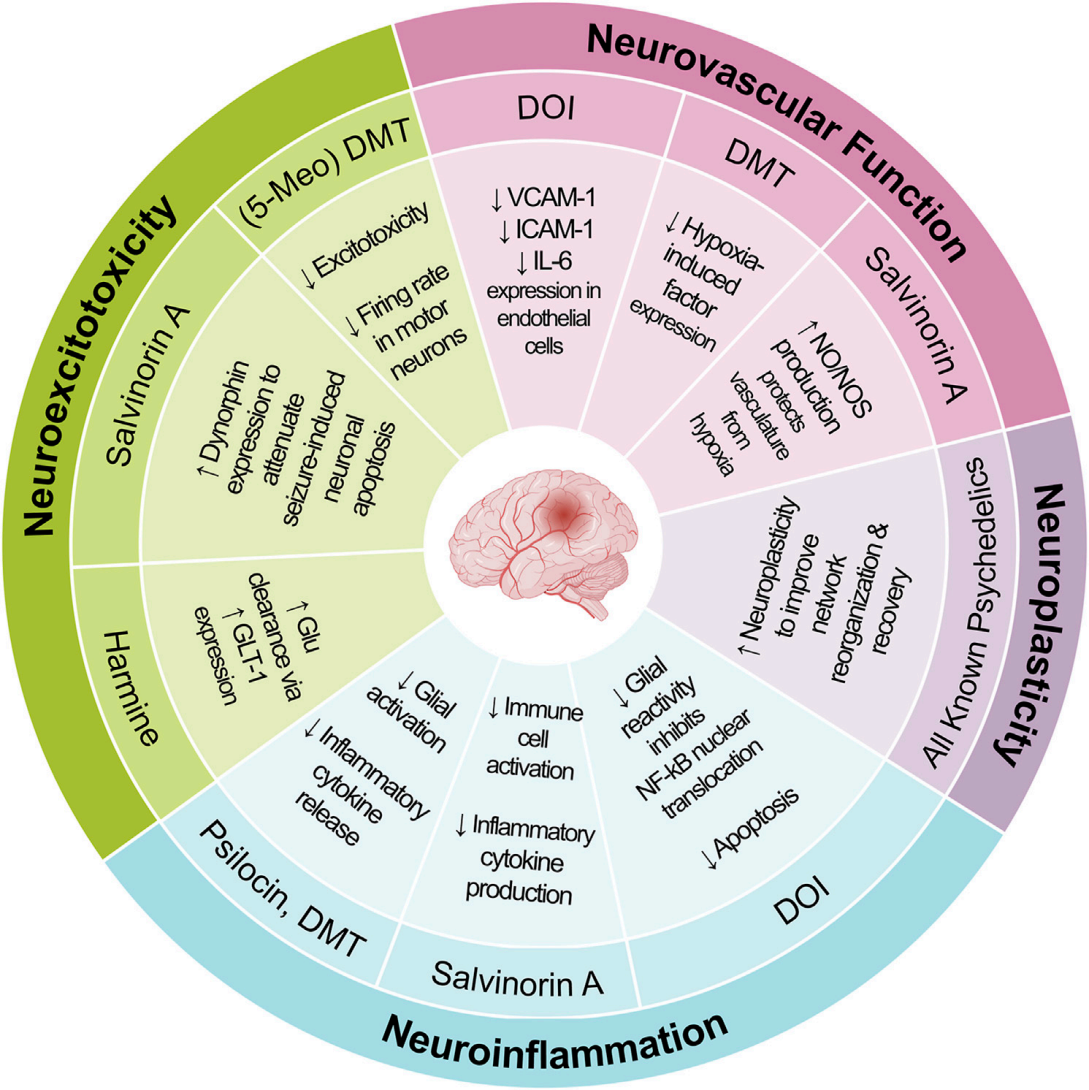
Review of possible mechanisms of action by which various psychedelic compounds may attenuate the pathophysiology of and enhance recovery from brain injury disorders.

Brain injuries, e.g., stroke and traumatic brain injury (TBI), are a major contributor to mortality, and the socioeconomic burden on the global healthcare system exceeds $200 billion. TBI and stroke have similar chronic cellular and molecular pathological sequelae, such as dysfunctional immune, endocrine, and neurovascular function, as well as neuronal loss and white matter degeneration (Rabinowitz and Levin, 2014; Rafols, 2015; Campbell et al., 2019). Neuroplasticity is the major mechanism of functional recovery for both injuries, as well (Nudo, 2013; Hara, 2015). While hundreds of pharmacotherapies have been identified in experimental stroke models, only one has been translated into successful clinical use. Research in TBI has similarly failed to produce novel therapies, in fact, over 40 pharmaceuticals have failed in either phase II or III in the last 30 years. The capacity for psychedelics to modulate a wide variety of physiological, as well as psychological processes, that are impacted by neurotrauma warrants serious investigation (Figure 9). For example, psychedelics can increase BDNF expression and activate TrkB signaling, which has been shown *in vivo* to be neuroprotective, attenuate inflammation, induce neural plasticity, and normalize neural activity in models of stroke and TBI (Wurzelmann et al., 2017; Hutten et al., 2021). Ibogaine deserves specific attention in the context of improving recovery from brain injury, as it increases growth factor levels, e.g., GDNF, for 2–3 days after ingestion (Marton et al., 2019).

#### 4.2.1 Traumatic brain injury

TBI is caused by a discrete biomechanical event inducing physical trauma termed the *Primary injury*, and subsequently initiates evolving biochemical cascades referred to as the *Secondary injury* which is characterized by persistent glial reactivity, immune cell infiltration, metabolic deficits, excitotoxicity, BBB disruption, mitochondrial dysfunction, and programmed cell death, amongst other facets. Furthermore, this secondary injury phase is also associated with an increased risk of seizures due to heightened neuronal excitability and altered neurotransmission (Golarai et al., 2001). Over time, these secondary injury processes can culminate in post-traumatic epilepsy (PTE), a chronic condition characterized by recurrent seizures, further complicating the long-term management and prognosis of TBI patients (Mosini et al., 2020).

Endothelial cells increase surface expression of VCAM-1 and ICAM-1, which increase the attachment and infiltration of monocytes and neutrophils to release more pro-inflammatory cytokines and chemokines. Notably, stimulation of 5-HT_2A_ receptors with picomolar concentrations of DOI can reduce ICAM-1, VCAM-1, and IL-6 expression in rat aorta smooth muscle cells (Yu et al., 2008; Nau et al., 2013). This anti-inflammatory action is mediated by inhibition of NF-kB (a transcription factor) nuclear translocation. Translocation of NF-kB is a central step in astrocyte and microglia reactivity (i.e., transition to a pro-inflammatory phenotype), as well as the upregulation of cell adhesion molecules (e.g., ICAM-1) in endothelial cells. It is possible 5-HT_2A_ mediated inhibition of inflammation on smooth muscle cells and endothelial cells could attenuate BBB breakdown following TBI. Similarly, serotonergic psychedelics may be able to attenuate astrocyte and microglia reactivity. In fact, through a 5-HT mediated process psilocin and DMT are capable of attenuating LPS induced microglial reactivity *in vitro*, and subsequently improve survival of neurons when co-cultured with LPS treated microglia (Kozłowska et al., 2021). Of note, psilocin and DMT also reduced microglial expression of Toll-like receptor 4 (TLR4), which is transmembrane protein essential in detection of damage associated proteins and driving reactivity (Kozłowska et al., 2021). Further studies need to examine the significance of these effects in the context of TBI, however, these results further suggest a therapeutic potential for serotonergic psychedelics.

Another major facet of TBI is the highly elevated concentrations of extracellular glutamate, which can trigger cytotoxic intracellular Na^+^ and Ca^2+^ influxes. Elevated intracellular [Ca^2+^] causes mitochondrial dysfunction, which subsequently impairs ATP production and increases concentrations of oxidative reactive oxygen species (ROS) (Kokiko-Cochran and Godbout, 2018). Sig-1 receptors are chiefly expressed on the mitochondria-associated endoplasmic reticulum and facilitates stress responses by increasing intracellular levels of anti-stress and antioxidant proteins. Furthermore, Sig-1 receptor activation has also been shown to suppress microglial activation through Ca^2+^-dependent mechanisms, decreasing the release of inflammatory cytokines (Smith and Su, 2017). In models of TBI, activation of Sig1Rs has also been shown to reduce lesion volume, improve behavioral outcomes, and attenuate microglial activation (Do et al., 2016). DMT activation of Sig-1 receptors has been shown to decrease HIF-1alpha expression in hypoxic conditions, and independently inhibit downstream VEGF expression, in iPSC-derived cortical neurons and microglia-like immune cells *in vitro* (Szabo et al., 2016). This study also found that these effects directly mediated enhanced cell survival in hypoxic conditions. DMT and 5-MeO-DMT stimulation of Sig1 receptors has also been shown to inhibit pro-inflammatory cytokine production and T-cell priming capacity of monocyte-derived dendritic cells (Szabo et al., 2014). Given that Sig-1R agonists have been shown to attenuate stimulation has also been shown to attenuate excitotoxicity and reduce the firing rate in motor neurons *in vitro*, compounds like DMT and 5-MeO-DMT may also provide neuroprotective effects against excitotoxicity following TBI.

Brain injuries often involve ischemia, during which the sudden deprivation of oxygen and glucose can trigger a spreading depolarization (SD), i.e., a slow recurrent wave of intense neuronal and glial mass-depolarization of depolarization, which can propagate across the brain (Chung et al., 2016). SDs also induce a large metabolic demand, i.e., consumption of glucose and increased oxygen utilization, which exacerbates neuronal damage and inflammatory processes in tissue that is already nutrient and oxygen deprived (Menyhárt et al., 2017). Notably, in a rodent model of cerebral forebrain ischedmia with recurrent pharmacological induction of SDs, DMT pre-treatment was shown to decrease the amplitude, rate, and cumulative duration of SDs, and in turn promote neuron and astrocyte survival (Szabó et al., 2021). This study also found that Sig-1Rs, but not 5-HT receptors, were necessary for the attenuation of SD amplitude, rate, and duration.

Select psychedelics may also be capable of modulating the functionality of glial cells, whose normal functions are highly altered following TBI. For instance, harmine has been shown *in vivo* to exert neuroprotective effects following TBI. Specifically, harmine seems to attenuate inflammatory signaling and neurotoxicity in the hippocampus by increasing expression of astrocytic glutamate transporter 1 (GLT-1) to levels comparable to non-injured animals by 5 days-post-injury (Zhong et al., 2015). Improved neuronal survival and attenuated neuronal excitability may have also mediated the reported improvements in learning and memory (Zhong et al., 2015). Psilocin and DMT have also been shown to modulate functionality of activated microglia. Specifically, both compounds seemed to attenuate microglial phagocytosis and inflammatory signaling, which subsequently improved neuronal survival in microglia-neuron cocultures (Kozłowska et al., 2021). Overall, these findings suggests a therapeutic potential of an ayahuasca brew with a potentially optimal concentration of harmine and DMT. Indeed, the synergistic potential of these compounds in the context of TBI should be investigated.

TBI can modulate seizure thresholds and predispose networks to hyperexcitability via multiple mechanisms, such as loss of inhibitory neurons, enhanced excitatory connectivity and glutamate release, and axonal sprouting. Dynorphins, a family of endogenous neuropeptide KOR ligands, play an important role as regulators of neuronal excitability, and thus epileptic activity. In fact, KOR stimulation has been shown to protect against seizure-induced neuronal apoptosis and inflammatory signaling. Also, salvinorin A administration following 6-Hz psychomotor stimulation and pentylenetetrazol injection acute seizure paradigms in a non-diseased mouse model has been shown to have no impact on seizure threshold activity. However, these epilepsy models fail to recapitulate the relevant pathophysiology and are not as informative for post-traumatic epilepsy (PTE) prevention (Mosini et al., 2020; Socała et al., 2020).

In the days and weeks following TBI, cortical and hippocampal neurons exhibit dendritic fragmentation, decreased dendritic branching, and a lower density of dendritic spines (Gao et al., 2011; Ratliff et al., 2020). Recovery from TBI is primarily driven by endogenous neuroplasticity mechanisms. Psychedelics can increase dendritic arborization complexity, spinogenesis, and synaptogenesis, which may help attenuate cognitive deficits associated with TBI-related dendritic and spine degeneration, as well as increase the ceiling for and rate of recovery. The ability for psychedelics to induce plasticity is mediated through activation of BDNF’s high-affinity receptor TrkB and downstream mTOR signaling. In fact, BDNF levels are also increased in the 24 h after TBI as an endogenous neuroprotective response, and administration of BDNF in brain has been shown to significantly attenuate TBI-induced neurological and cognitive (Wurzelmann et al., 2017). Although, BDNF’s short-half life and BBB impermeability renders it clinically unviable, direct stimulation of the high-affinity BDNF receptor, TrkB, has been shown to produce comparable functional recovery (Krishna et al., 2018). So, the capacity for psychedelics to efficaciously promote neuroplasticity via activation of TrkB may promote functional recovery from TBI.

*In vitro* studies should utilize 3D biomimetic microenvironments capable of recapitulating the mechanical forces and injury dynamics of TBI, so that the neuroprotective and neurorestorative effects of psychedelics can be studied within a relevant injury context. For example, Bar-Kochba et al developed a 3D *in vitro* compression model of TBI that could generate distinct levels of injury severity (Bar-Kochba et al., 2016). Alternatively, our group has utilized an electro-mechanical cell shearing device that can reliably reproduce biomimetic strain fields as a heterogeneous function of tensile, compressive, and shear strains generated within 3D cell cultures (LaPlaca et al., 2005; Cullen and LaPlaca, 2006; Cullen et al., 2007; Bar-Kochba et al., 2016). Nonetheless, animal models, typically rodents, are also crucial in preclinical TBI research. Controlled cortical impact (CCI) and lateral fluid percussion injury (LFPI) paradigms are both standard in TBI research for modelling focal injury and diffuse injury respectively (Cullen et al., 2016). These animal models would be ideal for assessing the capacity of psychedelics, administered at various timepoints relative to the injury, to attenuate inflammation and improve recovery following TBI.

If psychedelics can attenuate a chronic pro-inflammatory state, improve BBB integrity, and potentiate neuroplasticity, they could have pharmacological utility in the treatment of TBI. Indeed, there is evidence from *in vivo* ketamine TBI studies that indicate that some modes of action for psychedelics may serve to attenuate chronic damage and enhance functional recovery (Peters et al., 2018). Comparative assessments of the efficacy of different psychedelic compounds, acute *versus* chronic drug administration, and various timings for starting pharmacotherapy should be the focus of future studies.

#### 4.2.2 Stroke

Stroke is an acute cerebrovascular injury caused by an event that decreases blood flow to the brain leading to ischemia (Wurzelmann et al., 2017). Ischemia can impair artery autoregulative function, induce neuronal loss, cause inflammation, and impair BBB permeability, leading to chronic complications. Treatment focuses on restoring blood flow using thrombolysis (via administration of tissue plasminogen activator), or in more severe cases thrombectomy, to minimize extent of acute ischemic injury; however, only a relatively small portion of the patient population can receive these therapeutic interventions (Wu et al., 2020). There is a pressing need for pharmacotherapy that can improve blood flow immediately after injury, while also attenuating chronic inflammation and neurological dysfunction. Unfortunately, while thousands of therapeutic compounds and strategies have been successfully tested in animal models, none have translated into clinical care settings.

Administration of salvinorin A has been shown to preserve artery autoregulative function, reduce infarct size, and protect BBB integrity in piglet models of ischemic stroke (Su et al., 2012; Wang et al., 2012; Chen et al., 2014; Grothusen and Liu, 2019). Moreover, salvinorin A is an extremely potent vascular dilator, even under hypocarbia and endothelin induced vasoconstrictive conditions (Su et al., 2011). Salvinorin A preserves cerebrovascular function in part by stimulating nitric oxide (NO) production and activation of endothelial nitric oxide synthase (NOS). Endogenous elevation endothelial NOS levels in microvasculature following stroke injury are thought to confer a protective effect from secondary injury, so it is possible salvinorin A is enhancing this endogenous mechanism. Acute intranasal administration of salvinorin A was also shown to reduce infarct volume and improve neurological outcomes in a rhesus monkey ischemic stroke model (Wu et al., 2020). Salvinorin A might be most advantageous administered immediately after injury, while patients await pre-surgical CT scans, because it will immediately restore blood flow and could provide neuroprotective effects.

Following stroke, microglia and infiltrating immune cells, e.g., macrophages and lymphocytes, produce an exacerbated immune response that causes chronic complications. Cannabinoid receptor 1 (CB1) is expressed in neurons and immune cells, and its activation has been shown to produce neuroprotective effects in ischemia. Although salvinorin A is often referred to as a selective KOR agonist, computation studies have shown a high docking efficiency between salvinorin A and CB1. Moreover, salvinorin A stimulation may induce the formation of functional heterodimers between the two receptors (Xu et al., 2016). In fact, salvinorin A activation of CB1 signaling has been shown to be necessary for mediating various behavioral effects and physiological effects across animal models (Braida et al., 2007; Fichna et al., 2009). For instance, salvinorin A at picomolar concentrations has been shown to inhibit LPS-stimulated macrophage production of nitrite, TNF-alpha, and IL-10, by activation of KORs and CB1 receptors (Aviello et al., 2011)**.** The distinct contributions and synergistic relationship between KORs and CB1 receptors merit detailed investigation and may inform the ongoing development of salvinorin A analogs.

Stroke is often modelled *in vitro* by inducing ischemia-like conditions via an oxygen and glucose deprivation paradigm, which can be readily and simply applied to most tissue models. One notable model for stroke research is the commercially available Flocel DIV-BBB3D system which supports a 3D co-culture of brain endothelial cells and astrocytes, while also providing flow and permitting investigation of leukocyte transmigration (Holloway and Gavins, 2017). 3D multi-cellular models such as this would be ideal for more refined assessments of how salvinorin A protects BBB integrity, and how distinct cell-types mediate this effect.

Although there is a plethora of *in vitro* and *in vivo* injury paradigms, it is crucial to identify functional measures that reflect human brain injury-induced deficits. For example, stroke patients often experience cognitive fatigue and attention deficits during recovery (Johansson and Rönnbäck, 2012; Barman et al., 2016). It is possible that neuroplasticity may drive recruitment of larger and more diffuse networks, which is computationally expensive and impairs cognitive flexibility. Here, Hosking et al. developed a rodent model of willingness to expend cognitive effort, by which a rat can either engage a low-effort and low-reward task or a high-effort and high-reward task (Hosking et al., 2016). This model demonstrated that cortical network recruitment directly reflected willingness to engage in high-effort tasks. Alternatively, nest-building activity in mice following stroke is a multi-faceted functional measure of long-term sensorimotor and cognitive deficits (Yuan et al., 2018). This behavior is performed naturally and spontaneously, so it would be ideal for assessing any impairments to typical behavior and cognition. These models allow for contextually relevant assessment of stroke induced cognitive and behavioral impairment and can help assess whether psychedelics might worsen or improve cognitive processing, fatigue, and behavior in patients at different stages of recovery from brain injury.

#### 4.2.3 Psychedelic-assisted therapy for brain injury patients

It is crucial to consider that stroke and TBI patients are more predisposed to developing a psychiatric disorder, e.g., SUD, MDD, or PTSD (Kaplan et al., 2018; Ponsford et al., 2018). At present, it is unclear whether this elevated risk is due to the neuropathology of brain injury or due to psychological stress following injury. Regardless, it is possible the comorbid neuropathologies of brain injury and psychological disorders will modulate the efficacy and safety profile of psychedelic-assisted psychotherapy. For instance, it is unclear whether changes in blood pressure and cerebrovascular activity may introduce health risks in TBI patients. In fact, TBI patients are excluded from various ongoing clinical trials for psychedelic assisted psychotherapy. Notably, Davis et al surveyed US Special Operations Forces Veterans treated with ibogaine and 5-MeO-DMT for psychological trauma and found that most patients self-reported increased psychological flexibility, reductions in cognitive impairment, and reductions in symptoms of PTSD, depression, and anxiety, despite 82% of patients having sustained head injury (Davis et al., 2020). These results, and lack of adverse outcomes, may indicate that there are not elevated risks within the TBI patient population. Nonetheless, studies that control injury type and severity (e.g., mild, moderate, and severe) will be necessary to help assess the efficacy of psychedelic treatments for comorbid psychiatric conditions in TBI patients.

To determine the efficacy of psychedelics in comorbid patients, researchers should combine animal models of psychiatric disorders with TBI and stroke injury paradigms. For instance, Perez-Garcia et al. leveraged both PTSD paradigms and a rat model of TBI to demonstrate that rats exposed to low-level blasts were more vulnerable to the predator scent paradigm. Yet, blast-exposure could induce PTSD related traits in the absence of a physiological stressor (Perez-Garcia et al., 2016; Perez-Garcia et al., 2019). While rat TBI models are often scientifically relevant, they are generally not translational, due to poor replication of the head injury biomechanics (i.e., head rotational-acceleration), brain mass, and neuroanatomy (i.e., gyrencephalic with large white matter domains) that are drivers of TBI in humans. Accordingly, our lab utilizes a porcine model of head rotational acceleration, which has been shown to produce the same tissue deformation patterns, recovery delays, neuropathology, and inflammation profiles implicated in human TBI (Cullen et al., 2016; Wofford et al., 2021; Grovola et al., 2023). This injury paradigm could be applied in conjunction with a porcine psychosocial chronic stress model that assesses the development of a depressive phenotype using behavioral, endocrine, and gut biomarkers. Researchers have already begun to utilize the porcine model and characterize changes in gene expression following a single dose of psilocybin (Donovan et al., 2021). Together, these models will allow researchers to examine the therapeutic potential for psychedelics, as well as uncovering possible adverse side effects and risks for TBI patients.

### 4.3 Psychedelic interactions in the gastrointestinal tract

The GI tract regulates gut motility, local sensory processing, epithelial and immune function, appropriate microbiota responses, appetite, metabolism, and CNS function via the “gut-brain axis” described earlier (Morais et al., 2021). Absorption and digestion-related roles can also extend to the coordination of defense processes, like diarrhea, constipation, and vomiting, to eliminate toxic or indigestible substances or pathogenic microbes. Collectively, this wide range of functions has major implications for various to different aspects of clinical psychedelic administration. This includes identifying patients who do not respond to psychedelic therapy (“non-responder patient populations”), strategies to minimize potential harm (“harm reduction”), and ways to optimize therapeutic outcomes (“benefit maximization”).

#### 4.3.1 Intestinal inflammation

Inflammatory bowel disease (IBD) is a chronic and debilitating multifactorial disease that has increased with industrialization and results in over $8 billion in healthcare-related costs annually in the United States alone (Alatab et al., 2020). It is generally characterized by inflammation of the intestine (ulcerative colitis) or any part of the GI tract (Crohn’s disease), with 20%–50% of patients displaying behavioral disturbances, e.g., depression, anxiety, and visceral pain, that can predict relapse and disease severity (Bisgaard et al., 2022). Interestingly, nonsteroidal anti-inflammatory drugs and immunosuppressant therapy are commonly prescribed to patients and can sometimes reduce behavioral symptoms. Conversely, cognitive behavioral therapy, mindfulness-based therapy and hypnotic therapy have moderate success for some IBD patients in conjunction with dietary interventions (Ehrmann et al., 2020). Most treatment options for this disease, however, do not “cure” it but rather serve to manage symptoms. Relatedly, disorders of gut-brain interaction, mainly irritable bowel syndrome (IBS) are a significant global health burden, affecting 40% of the population, and are characterized by any combination of motility disturbance, visceral hypersensitivity, altered mucosal and immune function, altered gut microbiota, and altered CNS processing (Keefer et al., 2022). IBS is often associated with dysregulated serotonin signaling, which regulated gut motility; patients fall into diarrhea, constipation, or diarrhea and constipation subtypes.

**FIGURE 10:**
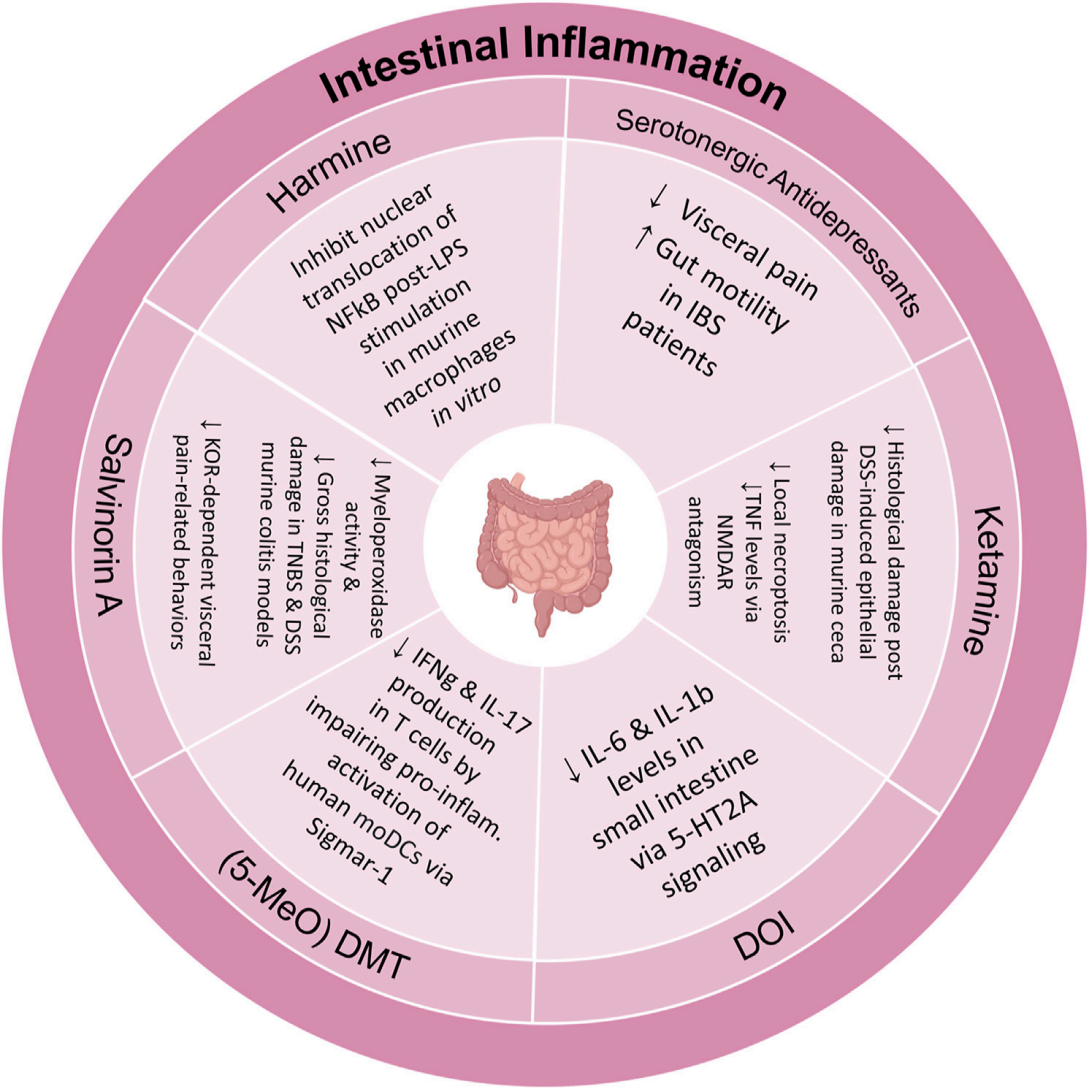
Review of possible mechanisms of action by which various psychedelic compounds may attenuate intestinal inflammation.

In non-comorbid settings, antidepressants are emerging as possible therapies for IBD and serotonergic drugs are current treatments for IBS (Ehrmann et al., 2020). Patients with IBS receiving antidepressant treatment reported benefits with visceral pain and gut motility (Ehrmann et al., 2020). Similarly, several psychedelics have demonstrated *in vivo* therapeutic effects in attenuating intestinal inflammation. Ketamine reduced histological damage after dextran sodium sulfate (DSS) treatment, a chemical method to induce epithelial damage in the ceca of mice and reduced local necroptosis, an inflammatory cell death, as well as TNF levels via NMDAR antagonism (Fujita et al., 2021). DOI, a psychedelic demonstrating the greatest efficacy in attenuating peripheral inflammation of any approved therapeutic, was able to reduce IL-6 and IL-1b levels in the small intestine, but not the colon, via 5HT2A signaling (Nau et al., 2013; Nau et al., 2015). DMT and 5Meo-DMT reduced IFNg and IL-17 production in T cells *in vitro* by impairing pro-inflammatory activation of human monocyte derived dendritic cells via Sigmar-1 (Szabo et al., 2014). Salvinorin A reduced myeloperoxidase activity as well as gross histological damage in both TNBS and DSS models of colitis that were accompanied by reduced visceral pain-related behaviors in mice in a KOR-dependent manner (Fichna et al., 2012; Sałaga et al., 2014). Finally, harmine inhibited nuclear translocation of NFkB after LPS stimulation in murine macrophages *in vitro* (Liu et al., 2017). Notably, anti-inflammatory action was not correlated with antidepressant or anxiolytic activity and the doses used in these studies were sub hallucinogenic (Flanagan and Nichols, 2022). In sum, it is highly plausible that multiple psychedelics, in combination with therapy and dietary interventions, could alleviate clinical GI symptoms present in both IBD and IBS-type populations through various mechanisms. We provide an overview of the possible mechanisms by which psychedelic compounds may modulate intestinal inflammation in Figure 10. An important consideration is the heterogeneity of patients with these diseases; different psychedelics will not be effective for all clinical presentations and could be potentially harmful for some (e.g., IBS-diarrhea patients), thus more *in vivo* mechanistic preclinical studies are needed to understand how they work in the intestine and GI tract more broadly (Nichols, 2022).

#### 4.3.2 Side effects

Common side effects of oral drugs, including psychedelics like ayahuasca, ibogaine, and psilocybin mushrooms, include nausea, vomiting, diarrhea, and constipation. Generally, nausea and vomiting processes are carried out by afferent vagal nerve signals from the gut to different brain regions, with a major role for 5-HT_3_, an ion channel receptor, whereas diarrhea and constipation are limited to the GI tract, specifically epithelial tight junctions (Mawe and Hoffman, 2013). While the exact mechanism behind these side effects is not known, it is hypothesized that serotonin agonism by their psychoactive counterparts: DMT, noribogaine, and psilocin, respectively, in the intestine is the main driver, with a smaller contribution of non-psychoactive compounds like harmine in ayahuasca and non-digestible chitin in psilocybin mushrooms. However, this does not fully explain how or why side effects vary widely among individuals and whether this correlates to physiological or psychological changes in the brain. Additionally, MDMA-induced hyperthermia in rats is attenuated by antibiotic treatment, concurrent with a bloom of the bacterium *P. mirabilis* in non-antibiotic treated rats supporting the mediating role of the gut flora in mitigating dangerous side effects (Ridge et al., 2019).

Enzymes that inactivate psychedelic compounds or that process pro forms of psychoactive compounds are often expressed in the stomach, liver, and intestine. Intestinal alkaline phosphatase, the enzyme responsible for dephosphorylating psilocybin to psilocin, is a common marker for differentiated intestinal epithelial cells. People with nonfunctional genetic mutations in cytochrome p450 genes, expressed in the liver and intestine, have higher serum levels of LSD at least 24 h post administration (Vizeli et al., 2021). Moreover, individual features of the GI milieu, such as the gut microbiota and host genetics may underlie differences in psychedelic efficacy and side effects.

#### 4.3.3 Intestinal epithelium

While many studies have looked at direct immunological effects, either *in vitro, in vivo* systemically, or *in vivo* in the intestine, studies have overlooked the effects of psychedelics on all other cell types in the intestine, most of which express serotonin receptors, for example, (Mawe and Hoffman, 2013). In terms of orally ingested psychedelics, the intestinal epithelial barrier will present first interaction *in vivo* and so could be a good research target to study. Kim and Ingber et al developed a microfluidic “Gut-on-a-Chip” technology where human Caco-2 epithelial cells are exposed to physiological peristalsis-like motions to induce self-organization of cells into 3D intestinal villi (Kim and Ingber, 2013). Another model, functional organoids, generated from intestinal stem cells from human patients may also present a unique *in vitro* opportunity to study intestinal inflammation and side effects although integration with other systems (microbial, immune, and neuronal) will require further optimization (Min et al., 2020). Gut-on-a-chip technology is ideal to assess the impact of psychedelics on intestinal inflammation under different conditions (e.g., microbial colonization) and dissect mechanisms in a controlled manner not possible with existing *in vitro* or *in vivo* models, while incorporation of organoids are useful for their clinical relevance (Kim et al., 2012; Kim et al., 2016; Jalili-Firoozinezhad et al., 2019; Angus et al., 2020). Overall, microfluidics and organoid models could be novel and effective tools for researchers to investigate applications of psychedelics as potent immunosuppressive agents for the treatment of intestinal disorders like IBD and IBS.

## 5 Conclusion

Research into psychedelics not only offers the promise to greatly improve wellbeing across multiple patient populations but may fundamentally challenge and inform novel theories of consciousness, our understanding of psychiatric disorders, and how to treat them. We believe that an emphasis on preclinical *in vitro* and *in vivo* studies assessing the mechanisms of action across a wide repertoire of psychedelic compounds will help move the field towards this goal. These investigations may also inform further, unimagined clinical applications and guidelines for psychedelic use. In fact, there are other potential applications that merit consideration, including neurodegenerative disorders and neurotrauma, as well as the potential role of the enteric nervous system and microbiome. However, it is crucial to underline the importance of proper usage, backed by the comprehensive FDA approval process, with a keen emphasis on strong safety data, and a clear understanding of potential side effects prior to the prescription of these compounds for specific disorders. We must also understand how comorbid conditions, like TBI, may alter the efficacy and safety of psychedelic-assisted therapy. We hope this review, by summarizing the potential modes of action and novel therapeutic applications, serves to expand the horizon for future pre-clinical and clinical investigations with psychedelic compounds.
